# Engineering liquid metal-based nanozyme for enhancing microwave dynamic therapy in breast cancer PDX model

**DOI:** 10.1186/s12951-023-02121-9

**Published:** 2023-10-31

**Authors:** Qiong Wu, Yongnian Yu, Xiaorui Yu, Qijun Du, Li Gou, Longfei Tan, Changhui Fu, Xiangling Ren, Jun Ren, Kai Xiao, Xianwei Meng

**Affiliations:** 1 Laboratory of Controllable Preparation and Application of Nanomaterials, Technical Institute of Physics and Chemistry, Chinese Academy of Sciences, Beijing, 100190 China; 2 CAS Key Laboratory of Cryogenics, Technical Institute of Physics and Chemistry, Chinese Academy of Sciences, Beijing, 100190 China; 3 College of Biomedical Engineering, Sichuan University, Chengdu, 610065 China; 4 Sichuan Kangcheng Biotechnology Co., LTD, No.28 Gaopeng Avenue, High-tech Zone, Chengdu, 610000 China; 5 Precision Medicine Research Center & Sichuan Provincial Key Laboratory of Precision Medicine and National Clinical Research Center for Geriatrics, West China Hospital, Sichuan University, Chengdu, 610041 China

**Keywords:** Liquid metal, Zeolite imidazolate framework, Nanozyme, Microwave dynamic therapy, PDX model

## Abstract

**Backgrounds:**

The novel concept of microwave dynamic therapy (MDT) solves the problem of incomplete tumor eradication caused by non-selective heating and uneven temperature distribution of microwave thermal therapy (MWTT) in clinic, but the poor delivery of microwave sensitizer and the obstacle of tumor hypoxic microenvironment limit the effectiveness of MDT.

**Results:**

Herein, we engineer a liquid metal-based nanozyme LM@ZIF@HA (LZH) with eutectic Gallium Indium (EGaIn) as the core, which is coated with CoNi-bimetallic zeolite imidazole framework (ZIF) and hyaluronic acid (HA). The flexibility of the liquid metal and the targeting of HA enable the nanozyme to be effectively endocytosed by tumor cells, solving the problem of poor delivery of microwave sensitizers. Due to the catalase-like activity, the nanozyme catalyze excess H_2_O_2_ in the tumor microenvironment to generate O_2_, alleviating the restriction of the tumor hypoxic microenvironment and promoting the production of ROS under microwave irradiation. In vitro cell experiments, the nanozyme has remarkable targeting effect, oxygen production capacity, and microwave dynamic effect, which effectively solves the defects of MDT. In the constructed patient-derived xenograft (PDX) model, the nanozyme achieves excellent MDT effect, despite the heterogeneity and complexity of the tumor model that is similar to the histological and pathological features of the patient. The tumor volume in the LZH + MW group is only about 1/20 of that in the control group, and the tumor inhibition rate is as high as 95%.

**Conclusion:**

The synthesized nanozyme effectively solves the defects of MDT, improves the targeted delivery of microwave sensitizers while regulating the hypoxic microenvironment of tumors, and achieves excellent MDT effect in the constructed PDX model, providing a new strategy for clinical cancer treatment.

**Supplementary Information:**

The online version contains supplementary material available at 10.1186/s12951-023-02121-9.

## Introduction

Breast cancer is the most common malignancy in women [1]. As a particular organ, the treatment outcomes of breast cancer depend on simultaneously the oncological and cosmetic factors [2]. For decades, surgical resection was the standard treatment for breast cancer. The treatment has experienced an evolutionary process to breast conserving therapy from extended mastectomy [3–6], radical mastectomy [7], and modified radical mastectomy [8, 9]. The alterative minimal invasive and noninvasive techniques have brought light to breast conserving therapy, which is a preferred therapy over mastectomy.

Microwave (MW) ablation is a widely utilized minimally invasive treatment of tumor in clinics. As a kind of electromagnetic wave with long wavelength, MW is less susceptible to bone and gas interference, and thus has deeper tissue penetration than light and ultrasound [10–12]. As such, MW ablation is promising for local treatment of breast cancer, and particularly exciting for cosmetic improvements. However, MW ablation has heated and warm zones, and residual tumors in warm zones may lead to tumor recurrence after MW treatment, hindering its clinical application prospect in breast conserving therapy.

Reactive oxygen species (ROS)-based cancer therapy modality, such as photodynamic therapy [13–16], sonodynamic therapy [17–20], chemodynamic therapy [21–24], and radiodynamic therapy [25, 26], provides an effective approach for overcoming the heat endurance of residual tumor cells. Our group has also previously developed a novel ROS-based cancer therapy strategy, MW dynamic therapy (MDT), which takes advantage of MW and utilizes MW energy alone to yield ROS for eradicating cancer cells [11, 12, 27]. Even so, the effect of ROS-based therapeutic strategies including MDT is still limited by the amount of sensitive agents reaching the tumor region. Moreover, the hypoxic characteristics of tumor microenvironment (TME) also hinder the production of ROS, severely reducing the therapeutic efficiency [15, 28–31]. Thus, as an emerging ROS-based therapeutic strategy, rational design of a new MDT agent with not only selectively internalizing by the tumor cell to solve the problem of poor delivery of microwave sensitizers, but efficient O_2_ self-supplying performance to eliminate the obstruction of tumor hypoxic microenvironment is highly desirable.

Fortunately, with rapid advances in materials science, liquid metals offer the opportunity to attenuate MW energy, transferring electrons to the surrounding oxygen for generating ROS [11]. Additionally, liquid metals are reported to have good biosafety and fluid properties [32], can adapt to complex biological systems, and undergo intracellular deformation when stimulated by external energy [33, 34]. Moreover, liquid metals can also be used in a variety of biomedical imaging, including photoacoustic imaging, CT imaging, X-ray imaging, and MRI imaging, etc., which is expected to further expand into a biomedical delivery platform for integrated diagnosis and treatment [35, 36], showing great potential in the biomedical field. Due to these excellent physicochemical properties, liquid metals have broad application prospects in tumor MDT. However, liquid metal-mediated tumor MDT still needs to address the major concerns of targeted delivery and tumor hypoxia. Currently, various strategies have been employed to fight against the tumor hypoxia [19, 28, 37]. Catalyzing the excessive H_2_O_2_ in the tumor to generate O_2_ can not only regulate the TME, but also greatly augment the effect of MDT.

**Scheme 1:**
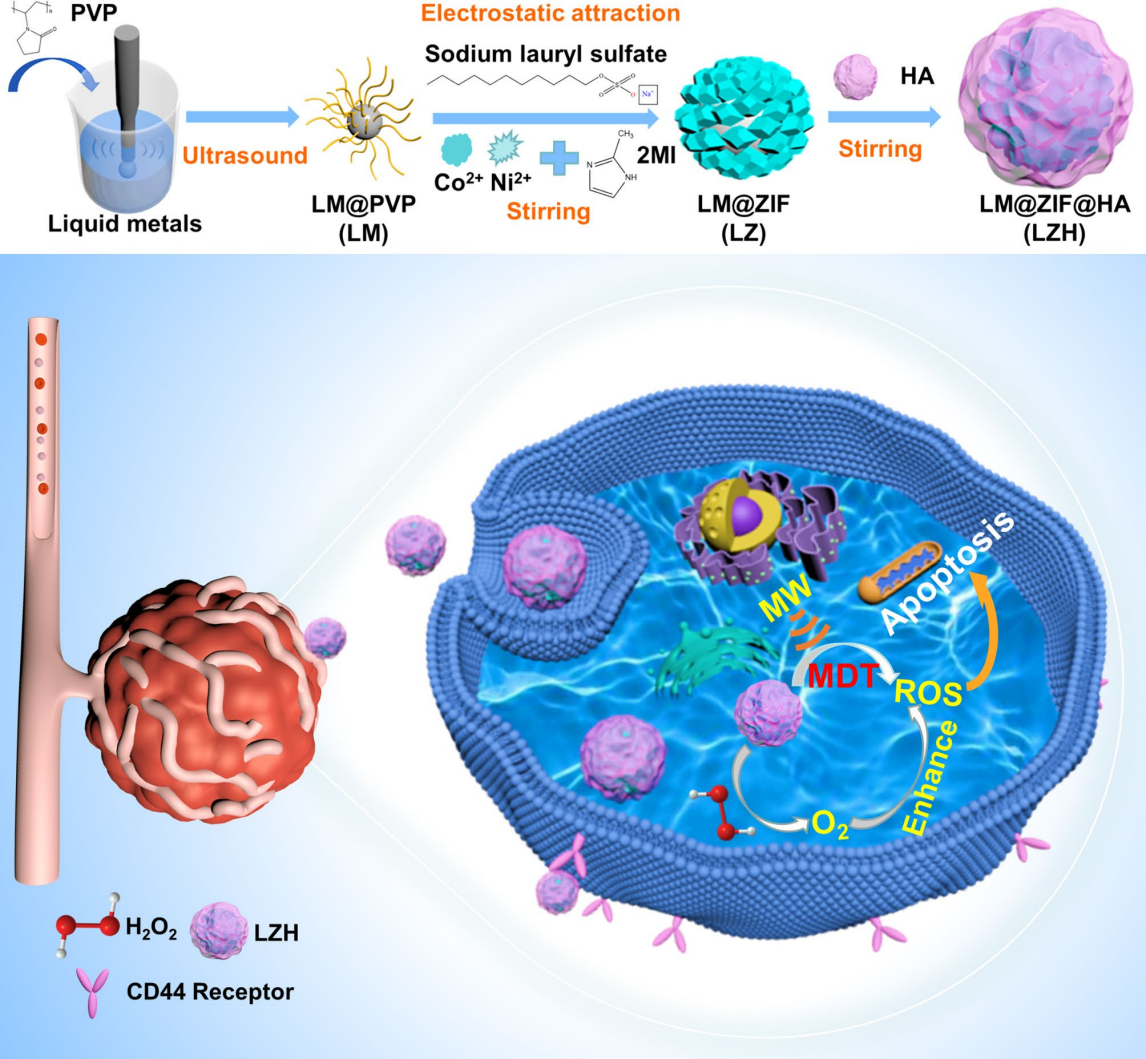
Schematic illustration of major steps of the preparation of LZH and overview of the LZH in tumor therapy

Accordingly, we first designed and constructed a targeted liquid metal-based nanozyme, in which the nanoscale liquid metal particles were coated by CoNi-ZIF and HA, called LM@ZIF@HA (LZH) with oxygen self-sufficient ability to promote MDT (Scheme 1). The targeting ability of HA and flexibility of liquid metal improve the accumulation of LZH nanozyme in tumor cells, which effectively solves the problem of poor delivery of microwave sensitizers. Catalase (CAT) -like activity can also catalyze the conversion of excess H_2_O_2_ in TME to O_2_, improve the hypoxic TME, and efficiently eliminate the obstacles of MDT. In addition, the nanozyme-mediated therapeutic process is often considerably hindered by the heterogeneity and complexity of the primary human tumor tissue [38–43]. Hence, a patient-derived xenograft (PDX) model of breast cancer is constructed to evaluate the treatment outcomes of the new therapeutic MDT agents with patient-like histological and pathological features. In the constructed breast cancer PDX model, the MDT of LZH nanozyme achieves excellent therapeutic effect, and the tumor inhibition rate is as high as 95%, showing great potential of the nanozyme for highly efficient tumor MDT.

## Materials and methods

### Materials

Cobalt nitrate hexahydrate (Co(NO_3_)_2_·6H_2_O, AR, 99%), polyvinyl pyrrolidone (PVP, K30) and 2-Methylimidazole (2-MI, 98%) were purchased from Shanghai Macklin Biochemical Co., Ltd. Nickel(II) nitrate hexahydrate (Ni(NO_3_)_2_·6H_2_O, AR, 99%) was provided by Beijing Chemical Plant. Sodium dodecyl sulfate (SDS) was supplied by Beijing Yili Fine Chemicals Co., Ltd. Hyaluronic acid (HA, 97%) was purchased from Shanghai Macklin Biochemical Co., Ltd. Rhodamine 6G was supplied by Tianjin Dye Industry Research Institute.

### Characterization and measurements

Zeta-potential and dynamic light scattering (DLS) measurements were tested using Zetasizer Nano series (Malvern ZEN3700,UK); SEM images were obtained by the scanning electron microscope (HITACHI S-4800, HITACHI, JAPAN); TEM images were gotten by the transmission electron microscope (HT7700, JEOL, JAPAN); Energy-dispersive spectroscopy was performed by JEM-2100 F transmission scanning electron microscope (JEM-2100 F, JEOL, JAPAN); X-ray diffraction pattern (PXRD) was obtained using the X-ray diffractometer (Bruker, D8 focus, Germany); DCF fluorescence was acquired by fluorescence spectrometer (Cary Eclipse, Shanghai, China); the surface functional group was measured by 3100 Fourier transform infrared spectrometer (FT-IR, Varian, USA); elastic modulus was tested using atomic force microscope (Bruker, Dimension FastscanBio, USA); enzyme standard instrument was used to the absorbance (EPOCH2, BioTek, Germany); the hyperspectral images were from Hyperspectral imager (Cytoviva, USA); fluorescence images of cell were obtained by Inverted fluorescent microscope (Nikon ECLIPSE Ti-S, Japan).

### Preparation of LM@PVP

First, 80 mg of PVP was added into 4 mL of deionized water and mixed evenly by ultrasonication. Then 62.5 mg of EGaIn was added into above mixture, and the large liquid metal droplets were transformed into small particles using ultrasonic crusher. The ultrasonic power was 400 W, the duration time was 3 s, the interval was 3 s, and total duration was 30 min. Afterwards, the upper suspension was collected by centrifugation at 10,000 rpm for 10 min. The precipitate was collected by centrifugation at 7500 rpm for 5 min and washed 3 times with ethanol for later use.

### Preparation of CoNi-ZIF

0.25 mol of Co(NO_3_)_2_·6H_2_O and 0.25 mol of Ni(NO_3_)_2_·6H_2_O were added into 4 mL of ethanol and mixed uniformly to prepare solution A. 21.9 mol of 2-methylimidazole (2MI) was dissolved in 9 mL of ethanol as solution B. And the solution C was prepared by mixing 40 mg of PVP with 2 mL of ethanol. Then, solution A was poured into solution B quickly. After the mixture was stirring for 2 min, solution C was added drop by drop and the final mixture was stirred magnetically for 12 h. The precipitate was collected by centrifugation at 8000 rpm for 5 min and washed with ethanol for 3 times.

### Preparation of LM@ZIF (LZ)

Since there are no active groups on the surface of LM and ZIF particles, they cannot interact to form core-shell structures. We tried to integrate ZIF with LM through electrostatic interaction. 0.0125 g of cobalt nitrate hexahydrate and nickel nitrate hexahydrate were dispersed in 4 mL of ethanol as solution A, 0.300 g of 2-methylimidazole and 0.018 g of sodium lauryl sulfate were dissolved in 9 mL of ethanol as solution B, and 2 mL of ethanol containing 0.040 g of PVP was made into solution C. Next, 9 mg of the above-synthesized LM@PVP was added to the B solution and stirred for 30 min. Then solution A was poured into solution B speedily, and solution C was dropped in with a dropper after 2 min. After 12 h, precipitation was obtained by centrifuging at 5000 rpm for 8 min.

### Preparation of LM@ZIF@HA (LZH)

In order to enhance the stability of LZ nanoparticles under physiological conditions, reduce its cytotoxicity, improve its biocompatibility and tumor targeting, HA was chosen to coat the MOF particles. 2 mg of HA powder was dissolved in 5 mL of deionized water and 10 µL of 1 M sodium hydroxide solution (NaOH) was added to adjust the pH value. Then, 10 mL of ethanol solution containing 10 mg of LZ was added to above solution. After stirring for 2 h, the precipitate was collected by centrifugation at 7000 rpm for 5 min and washed 2-3 times with ethanol.

### Load of rhodamine 6G

For the sake of proving the targeting of HA at the cell level and observing the amount of material endocytosis, Rhodamine 6G dye was added during the HA coating process to achieve the load of Rhodamine 6G. 2 mg of HA powder and 5 mg of Rhodamine 6G powder were dispersed in 5 mL of deionized water, and then 10 mL of ethanol solution containing 10 mg of LZ or ZIF was added and the mixture was stirred with magnetic stirrer for 2 h. Finally, the precipitate was collected by centrifugation and washed with ethanol for 3 times. The conditions of centrifugation were at 7000 rpm for 5 min.

### The catalase-like activity of LZ

2 mg of LZ was taken into 1 mL of deionized water. After the indication of dissolved oxygen analyzer, 10 µL of 0.1, 0.5, 1, 2 M hydrogen peroxide solution (H_2_O_2_) was added. Then recording the data every 30 s. Simultaneously, a hydrogen peroxide concentration was selected to conduct oxygen production tests with different material concentrations (0, 1, 3, 5 mg/mL). The hydrogen peroxide concentration was 7.5 mM. The data was recorded in the same way. Besides, 1 mg of LZ was added to 1 mL of deionized water. After the instructions of the dissolved oxygen analyzer, 5 or 10 µL of 10 mM H_2_O_2_ was added, and MW irradiation was applied simutaneously. The data was then recorded every 30 s.

### Endocytosis of LZH and ZIF@HA

4T1 cells were planted into a 6-well plate, and then 1 mL of 100 µg/mL LZH-Rhodamine 6G and ZIF@HA-Rhodamine 6G were added (the concentration of material was 50 µg/mL) to two of the wells. After 12 h, the residual material was washed away and the fluorescence was observed. Additionally, the fluorescence intensity was analyzed by flow cytometry.

### Hyperspectral imaging

10 µL of LZH nanozyme, ZIF@HA, and LM@PVP were dropped onto the glass slide, then the cover glass was covered on it. After the solution was dry completely, the hyperspectral microscope made by Cytoviva was used to observe the material. Similarly, the 4T1 cells were incubated with 50 µg/mL material for 12 h. Then the excess material was washed off with PBS and the cells were simply fixed with 4% neutral formaldehyde for 15 min. Afterwards, they were sealed and observed by hyperspectral microscope.

### ROS test in vitro

5 mg of LZ and 200 µL of DCFH-DA (0.5 mM) were dissolved in 2.3 mL PBS buffer solution (pH = 7.4). Then, 10 µL of 3% hydrogen peroxide solution was added into the mixture. After that, MW was irradiated for 10 min at power of 1.8 W. PBS and PBS + H_2_O_2_ were used as blank control. Afterwards, all the samples were kept in the dark for 2 h. Then, the precipitate was removed by centrifugation at 10,000 rpm for 5 min, and the supernatant was collected to measure the fluorescence of DCF (excitation wavelength: 480 nm, test wavelength: 500–600 nm, slit width: 2.5/5).

### Young’s modulus

10 µL of the same concentration of LM, ZIF, LZ were dropped on a clean circular mica plate, respectively. After ethanol was completely volatilized, mica plate was fixed to the flat round metal table. Then, Young’s modulus test was performed. Cantilever parameter: elastic coefficients k: 42 N/m, resonance coefficient f: 320 kHz, thickness T: 3.8 μm, length: 127 μm, width: 35 μm. The needle calibration radius 8–10 nm.

### Study of cytotoxicity in vitro

Murine mammary carcinoma (4T1) cells were used as the cell model. 4T1 and mouse fibroblast epithelial cells (L929) were incubated in complete medium composed of Dulbecco’s modification of Eagle’s medium (DMEM), 10% FBS, 1% antibiotics consisting of streptomycin and penicillin. 4T1 and L929 cells were cultured in a 96-well plate for 24 h, and then different concentrations of LZH nanozymes (0, 12.5, 25, 50, 100, 200 µg/mL) were incubated with these cells for 12 h, 5 parallel samples per group. Then 20 µL of MTT solution (5 mg/mL, i.e. 0.5% MTT) was added to each well. After 4 h, the culture medium was removed from the well and 150 µL of dimethyl sulfoxide (DMSO) was added to each well. After centrifugation (10,000 rpm, 5 min), 100 µL of supernatant was taken from each well and the absorbance was tested at 492 nm with a microplate reader. The cell viability was calculated by the absorbance and the cytotoxicity of the material was evaluated.

### Oxygen generation of LZH nanozyme

Tris(2,2’-bipyrimidine) ruthenium dihydrochloride [Ru(dpp)_3_]Cl_2_ (RDPP) was served as an oxygen probe. 4T1 cells were cultured with different concentrations of LZH nanozyme (0, 50, 100, and 200 µg/mL) for 12 h, and then 500 µL of 20 µg/mL RDPP was added (total volume: 1mL, that is, the probe concentration is 10 µg/mL). After 2 h, 500 µM of hydrogen peroxide was added and incubated for 3-4 h. Finally, the excess probes and hydrogen peroxide were washed away with DMEM and the fluorescence was observed through the fluorescence microscope.

### ROS test at cell level

LZH nanozyme at a concentration of 200 µg/mL was incubated with 4T1 cells in a well plate for 12 h. The medium in the wells was removed and the well was washed with PBS. Then, the digested cells were irradiated with MW at 1.8 W for 5 min. Next, these cells were incubated with DCFH-DA probes at a concentration of 10 µM. After 30 min, the excess probes were washed with PBS and the fluorescence was observed by fluorescent microscope. Additionally, the fluorescence intensity was analyzed by flow cytometry, and other experimental operations were completely consistent with the above.

### Targeting of HA

4T1 cells were incubated with 5 mg/mL of HA solution for 1 h, and the other group was supplemented with the same volume of medium as a control. Then the culture medium was detached and 50 µg/mL LZH-Rhodamine 6G was added. After 4 h, the excess material was washed away with PBS, and the fluorescence was observed through a fluorescence microscope. Moreover, the fluorescence intensity was analyzed by flow cytometry.

### Inhibition of 4T1 cells

50, 100, and 200 µg/mL of LZH nanozyme were incubated with 4T1 cells in the 6-well plate for 12 h. The control group was supplemented with the same volume of medium. And then the cells in each group were digested. The MW group was treated with MW irradiation at 0.9 or 1.8 W. After 5 min of irradiation, the cells were transferred to a 96-well plate and cultured for another 24 h. Then, 20 µL of MTT solution was added into each well and incubated for another 4 h. The excess culture medium was washed away and 150 µL of DMSO was added to the well. After centrifugation (10,000 rpm for 5 min), 100 µL of the supernatant was taken to test its absorbance at 492 nm. The cell viability was calculated by the absorbance.

### Live/dead staining assay

According to the results of the cell inhibition experiment, a material concentration of 100 µg/mL and a MW condition of 1.8 W for 5 min were selected to perform cell live/dead staining to evaluate the MDT of LZH nanozyme. 100 µg/mL of LZH nanozyme was incubated with 4T1 cells for 12 h, and then the digested cells were subjected to MW treatment. Then, the cells were stained with calcein-AM and propidium iodide (PI). After 30 min, the excess dye was washed with PBS and the fluorescence microscope was sued for observing the stained cells.

### Acute toxicity study

For evaluating the biosafety of the material, Balb/c mice of about 20 g were selected for the acute toxicity test. Mice were injected with different doses of materials (0, 50, 100, 200 mg/kg, dispersed in pH = 7.4 PBS buffer solution) via tail vein, 3 mice in each group. The state of the mice was observed carefully and changes in their weight were recorded every day. After 14 days, the mice were sacrificed and their blood was collected for blood routine tests. At the same time, their major organs including heart, liver, spleen, lung and kidney were collected. After slicing, fixing and staining, the tissue sections were observed with an optical microscope.

### Construction of PDX model

Firstly, nude BALB/c mice (7-8 weeks) were anesthetizing by 1.5% mebubarbit. Then, fresh tumor tissues were implanted subcutaneously into the right back of the mice. Fresh tumor tissue was obtained from breast cancer patient of the Department of Breast Surgery, West China Hospital, Sichuan University, with approval from the hospital’s biomedical ethics committee and in compliance with all relevant ethical regulations (2020 Review No. 353).

### In vivo experiment of LZH nanozyme

When bearing-mice in the PDX model were about 100 mm^3^, 20 mice were randomly divided into 4 groups: Control, MW, LZH, LZH + MW. Then the mice were subjected to tail intravenous injection (LZH and LZH + MW groups at a dose of 50 mg/kg, control and MW group injected the same dose neutral PBS). After 6 h, MW irradiation (1.8 W for 5 min) was performed on the group requiring MW irradiation. Subsequently, the weight of the mice and the size of the tumor had been recorded daily until the end of the experiment. After the experiment, mice were sacrificed, taking heart, liver, spleen, lung, kidney, and tumor. Finally, H&E staining and immunohistochemistry experiment were performed. Tumor volume = length × width × width/2-scar long × scar width × scar width/2. All animal experiments were conducted according to the Association for Assessment and Accreditation of Laboratory Animal Care guidelines and were approved by the Institutional Animal Care and Use Committee of West China Hospital, Sichuan University (file No.2,021,913 A).

### Statistical analysis

All results were expressed as the mean ± standard deviation (S.D). The analysis of statistical (*** P < 0.001, **P < 0.01, and *P < 0.05).

## Results and discussion

### Synthesis and characterization of LZH nanozyme

**Fig. 1:**
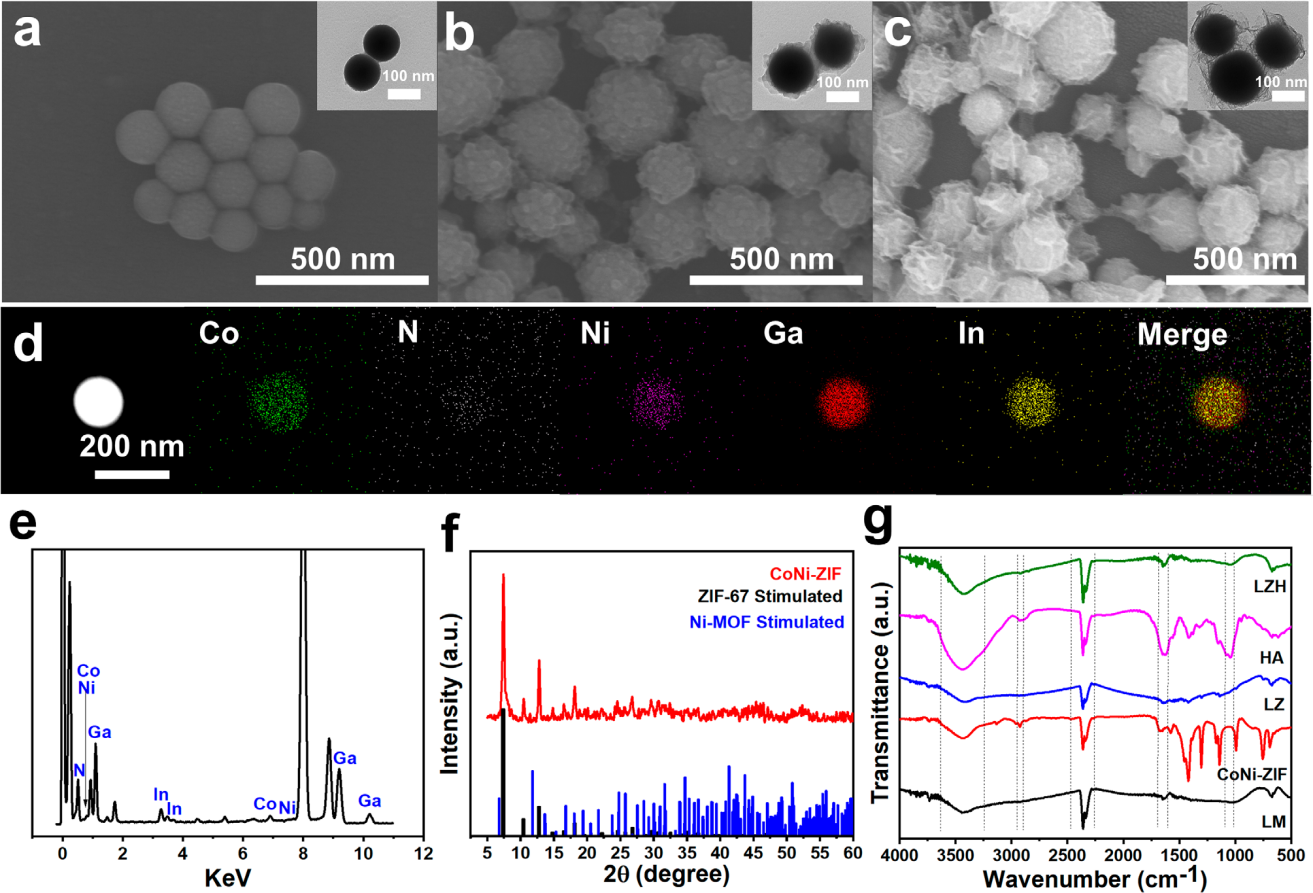
Characterization of LZH. (**a**) SEM and TEM images (interpolated) of nanosacle liquid metal LM@PVP NPs. (**b**) SEM and TEM (interpolated) images of LZ NPs. (**c**) SEM and TEM (interpolation) images of LZH. (**d**) EDS element mapping images of LZ NPs. (**e**) EDS elements of LZ NPs. (**f**) XRD spectra of CoNi-ZIF NPs and corresponding single metal MOF: simulated XRD spectra of ZIF-67 and Ni-MOF. (**g**) Fourier transform infrared spectra of LM@PVP, CoNi-ZIF, LZ, HA and LZH.

Although the high surface tension of liquid metal will be reduced after the rapid formation of a thin oxide layer on the surface under aerobic conditions, the problems such as uncontrollable interface and viscous oxide layer also hinder its application in the biomedical field. Herein, polyvinylpyrrolidone (PVP) with low biotoxicity was used as a surfactant to realize the nanometerization of bulk liquid metals through the top-down method by ultrasound. Spherical LM@PVP (LM) nanoparticles about 180 nm with smooth surface were observed, consistent with hydrated particle size measurement (Fig. 1a and Fig. S1a). Since there were no available groups on the surface of LM for binding with ZIF, it was hard to coat LM with ZIF via chemical reaction. Fortunately, LM was positively charged in the ethanol solution (Fig. S1d), so we introduced the anionic surfactant sodium lauryl sulfate. LM@ZIF (LZ) nanoparticles with an average particle size of approximately 250 nm were obtained by electrostatic attraction (Fig. 1b and Fig. S1b). After coating ZIF on LM, some small protrusions appeared on the surface, like the chocolate beans on cookies, which made the surface rougher than LM. The EDS mapping results of LZ nanoparticles showed that the Ga, In, Co, Ni, and N element presented spherical distribution, and the distribution of Co and Ni was obviously larger than that of Ga and In, which also proved the successful synthesis of LZ nanoparticles (Fig. 1d and e). X-ray diffraction pattern (XRD) results revealed that the peaks of the synthesized CoNi bimetallic MOF were basically consistent with the standard XRD patterns of pure Co and Ni MOF (Fig. 1f), confirming the synthesis of bimetallic ZIF. LZH nanozyme was formed by coating LZ nanoparticle with hyaluronic acid (HA) to improve its stability and confer targeting (Fig. 1c and Fig. S2). The absorption peak at 2960 cm^-1^ in Fourier infrared spectrum could be ascribed to the stretching vibration of -CH_3_ in the dimethylimidazole (Fig. 1g). The absorption of 1650 cm^-1^ was the bending vibration of N-H, and the absorption peak near 1050 cm^-1^ could be the stretching vibration of the C-O bond in HA. These results indicated the successful synthesis of LZH nanozymes. The hydrated particle sizes of LM, LZ and LZH were 180 nm, 250 nm, and 260 nm (Fig. S1c), and the corresponding PDI were 0.060, 0.101, and 0.119, respectively. The above results proved that the monodisperse LZH nanozymes were successfully synthesized.

### Evaluation of oxygen generation

**Fig. 2:**
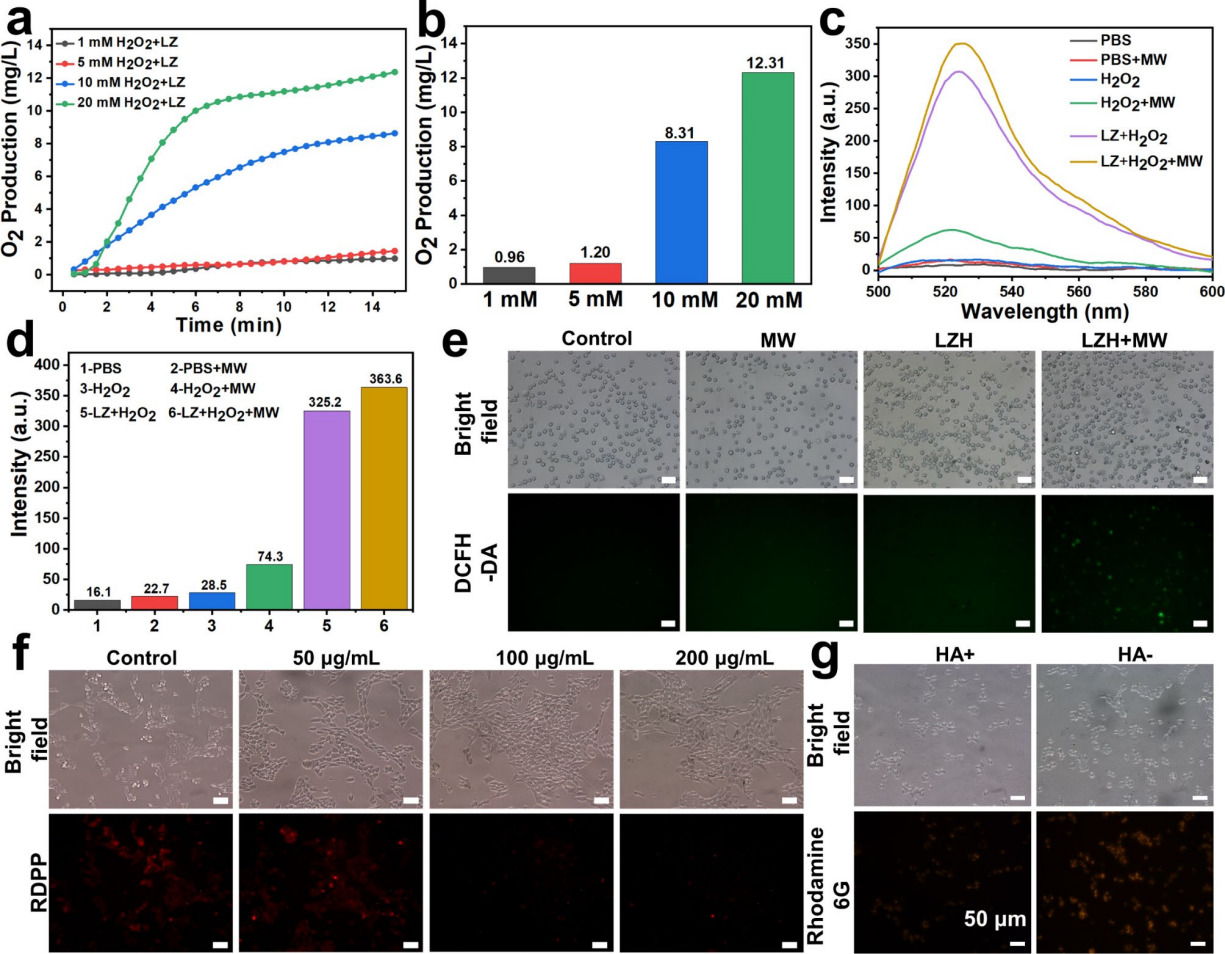
Physical and chemical properties. (**a**) The CAT-like activity of LZ NPs under different H_2_O_2_ concentrations (1, 5, 10 and 20 mM). (**b**) The net difference of oxygen production about a). (**c**) Fluorescence intensity of DCF in PBS under different treatments. (**d**) The peak fluorescence intensity of DCF in PBS under different treatments. (**e**) The fluorescence images of ROS production at the cellular level. (**f**) The fluorescence images of oxygen generation at the cellular level. (**g**) The fluorescence images of CD44 targeting performance

Researches have shown that liquid metals can produce ROS under MW irradiation [11]. However, the MDT can be negatively affected by hypoxia of TME. Hence, an oxygen self-sufficient agent is particularly important. The mechanism of reaction catalyzed by Co-based MOF is the conversion of H_2_O_2_ into O_2_ with the assistance of the Co^2+^ center in ZIF-67 [44]. Accordingly, the CAT-like activity of LZ nanoparticle was evaluated. When LZ nanoparticle was added to H_2_O_2_ solution, a violent reaction occurred immediately and a large amount of oxygen was generated, while H_2_O_2_ solution alone had almost no reaction (Fig. S4c and d). Next, CAT-like activity of LZ nanoparticle under different H_2_O_2_ concentrations was tested. When the concentration of H_2_O_2_ was 1 mM, the rate of oxygen generation was very slow, and the oxygen concentration at 15 min was also extremely small, only 0.96 mg/L (Fig. 2a). With the increment of the concentration of H_2_O_2_, the generation of oxygen gradually increased. At 20 mM, the amount of oxygen increasing within 15 min was about 12 times than that of 1 mM (Fig. 2b). In addition, to further demonstrate the CAT-like activity of LZ within tumors, we validated the CAT-like activity of LZ on H_2_O_2_ levels (50–100 µM) in tumors. The results showed that under MW irradiation, when the concentration of H_2_O_2_ was 50µM, the oxygen generation was 6.9 times that of the control group (Fig. S3). When the concentration of H_2_O_2_ reached 100 µM, the oxygen generation was 9.7 times that of the control group, and there were significant differences. Simultaneously, for further proving the catalytic ability of LZ, we also conducted corresponding experiments at different concentrations of LZ nanoparticles. Fig. S4a showed that the curve of the H_2_O_2_ solution without any treatment was relatively flat, indicating that the decomposition of H_2_O_2_ was very slow and the oxygen amount was pretty small (0.3 mg/L) without catalysis. While after adding LZ nanoparticles, the decomposition rate of H_2_O_2_ was significantly increased, even 1 mg could generate 4.41 mg/L of oxygen in 15 min. Moreover, with the increase of the concentration of LZ nanoparticles, the decomposition rate of H_2_O_2_ was also accelerated. They all tended to be flat around 15 min, which might cause by H_2_O_2_ depletion, but the oxygen generation of 5 mg at 15 min still reached 40 times that of the control group (Fig. S4b). All experiments above manifested that LZ nanoparticles had excellent CAT-like performance of H_2_O_2_ and was expected to enhance the production of ROS.

### In vitro study of ROS production

The redox in cells tend to a certain balance, and overexpression of either side will cause apoptosis. Therefore, ROS expression enhanced by external stimulation has great prospects in tumor therapy. Considering CAT-like performance of LZ and the MW dynamic effect of liquid metal, the ROS producting capacity of LZ nanoparticles was evaluated. LZ nanoparticles could produce a certain amount of ROS due to the existence of liquid metal, and MW stimulation could promote ROS production (Fig. S5). H_2_O_2_ effectively promoted ROS production, while LZ nanoparticles further significantly increased the level of ROS (Fig. 2c), which might be related to the CAT-like performance of LZ nanoparticles. The LZ + H_2_O_2_ + MW group had the highest ROS production, and the peak fluorescence intensity was 22.5 times that of the control group and 16 times that of the H_2_O_2_ group (Fig. 2d**)**. Next, ROS production of LZH nanozymes at the cellular level was further investigated. After incubating LZH nanozymes at a concentration of 200 µg/mL with 4T1 cells for 12 h, the MW group was irradiated with MW at 1.8 W for 5 min. Almost no green fluorescence was observed in the control group and the MW group **(**Fig. 2e), while very weak green fluorescence was observed in the LZH group. In contrast, the fluorescence of LZH + MW group was the strongest, revealing that LZH nanozymes could produce a large number of ROS in cells under MW stimulation. Fluorescence quantitative analysis was performed by flow cytometry, and the results were consistent with those observed by fluorescence microscopy (Fig. S6). The fluorescence of the LZH + MW group was 21.3 times that of the control group, 12.0 times that of the MW group, and 3.0 times that of the LZH group (Fig. S7). These results demonstrated that the LZH nanozymes could produce ROS efficiently and had CAT-like properties, which could promote O_2_ generation and lead to increased ROS production, laying a solid foundation for the enhanced MDT by oxygen self-supplying in vivo.

### Evaluation of oxygen generation at cellular level

The divalent ruthenium (Ru) complex excites to produce strong and stable red fluorescence, which can be effectively quenched by molecular oxygen. Accordingly, [Ru(dpp)_3_]Cl_2_ (RDPP) is often used as an oxygen probe to test the oxygen generation [30]. Given the excellent performance of LZH nanozymes in catalyzing H_2_O_2_ to generate O_2_in vitro, RDPP was used to further explore the oxygen self-supplying performance of LZH nanozymes at the cellular level. Bright red fluorescence can be seen in the control group (Fig. 2f), proving the hypoxia characteristics of 4T1 cells. As the concentration of LZH nanozymes increased, the red fluorescence gradually weakened. When the concentration was 200 µg/mL, the red fluorescence was barely visible, indicating that more oxygen was generated. Overall, LZH nanozymes had remarkable CAT-like properties and could realize intracellular oxygen self-supplying, thereby alleviating tumor hypoxia and enhancing tumor MDT.

### Targeting ability of LZH

Breast cancer cells are one of the cells with high expression of CD44 receptor, and HA can specifically target CD44 receptor, providing an effective approach for the treatment of triple-negative breast cancer [45, 46]. Considering the HA on the surface of LZH nanozymes, the targeting performance of LZH nanozymes was studied using “shielding” method. The experimental group was treated with HA solution first, while the control group did not do any treatment. After 2 h, the same concentration of LZH was added, and the fluorescence was observed after 4 h incubation. As shown in the Fig. 2g and S9, with the “shielding” of HA solution, the red fluorescence was significantly weaker than that of the control group. Further quantitative analysis by flow cytometry showed that the fluorescence intensity of cells without HA shielding was significantly increased (Fig. S10), which was 2.2 times that of the HA shielding group (Fig. S11). These results clearly suggested that LZH nanozymes had notable CD44 targeting performance and also proved that HA was successfully coated.

### Study on cellular endocytosis

**Fig. 3:**
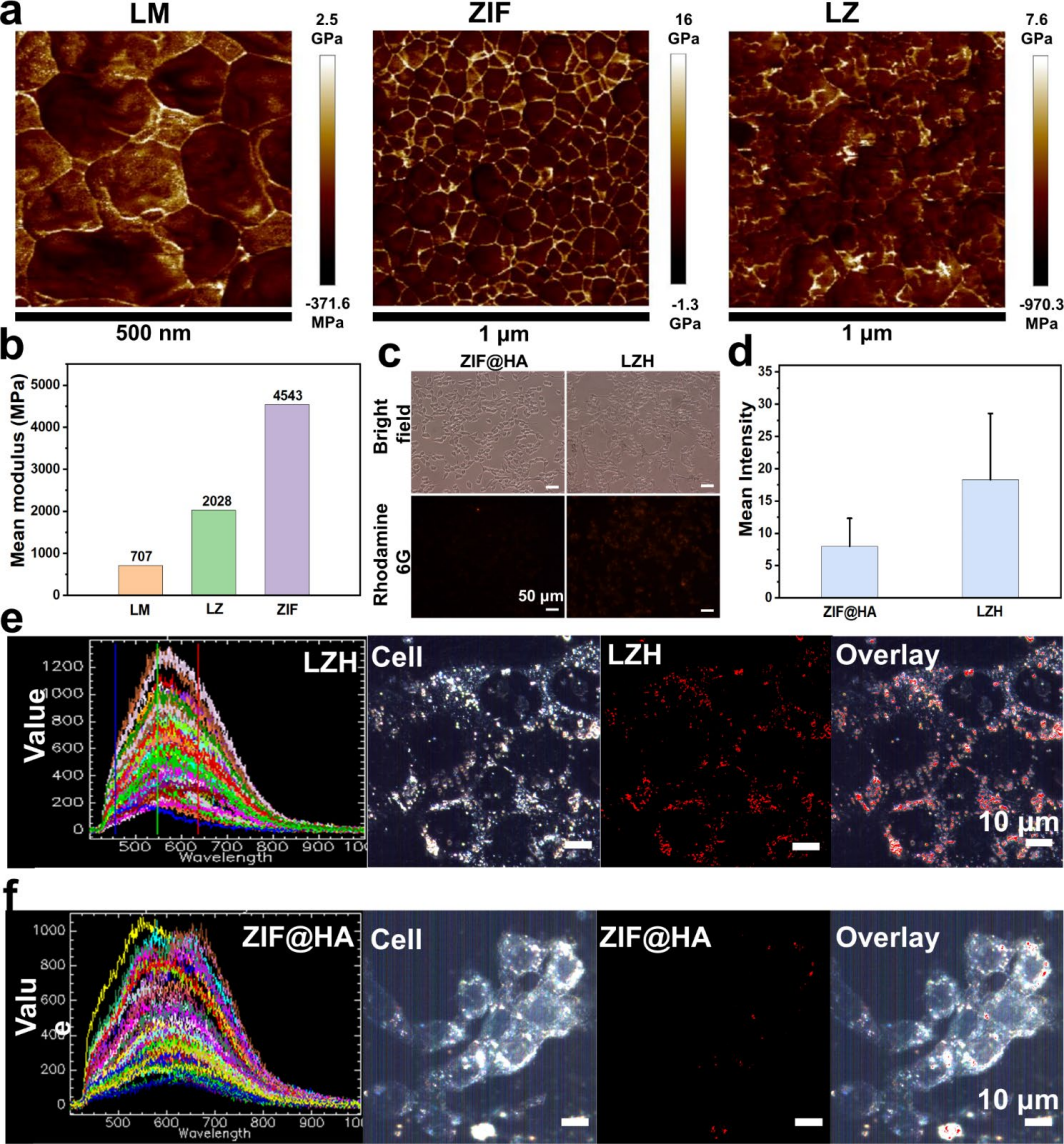
Endocytosis of ZIF@HA and LZH. (**a**) Quantitative nanomechanical mappings of LM, ZIF and LZ nanoparticles. (**b**) Averaged Young’s modulus of LM, ZIF and LZ nanoparticles. (**c**) Fluorescence image of Rhodamine 6G in 4T1 cells. (**d**) Mean fluorescence intensity of Rhodamine 6G in 4T1 cells. (**e**) Hyperspectral images of LZH nanozymes (From left to right: spectral library of LZH nanozymes, hyperspectral image of cells, mapping of LZH nanozymes uptaken by 4T1 cells, and overlay image of LZH nanozymes and cells). (**f**) Hyperspectral images of ZIF@HA (From left to right: spectral library of ZIF@HA, hyperspectral image of cells, mapping of ZIF@HA uptaken by 4T1 cells, and overlay image of ZIF@HA and cells)

Studies have shown that the flexibility of liquid metal allows it to deform, making it easier to uptake by cells [47]. Compared with ZIF, LZH nanozymes should be easier to enter into the cells, thus greatly enhancing the therapeutic effect of tumors. This hypothesis was verified by atomic force microscopy (AFM) detection (Fig. 3a). The average elastic modulus of LZ nanoparticles was 2038 MPa, much greater than LM (707 MPa) and smaller than ZIF (4543 MPa) (Fig. 3b). It was further verified by endocytosis assay. These nanoparticles were loaded with Rhodamine 6G to provide intracellular fluorescence tracking (Fig. S12). After incubating 4T1 cells with the same concentration for 12 h, the fluorescence was observed. The red fluorescence was stronger in the LZH group, but weaker in the ZIF group (Fig. 3c). The results of average fluorescence intensity showed that the fluorescence intensity of the LZH group was about twice that of the ZIF group (Fig. 3d), demonstrating that the endocytosis of LZH was much higher than that of ZIF, which was consistent with our hypothesis. Flow cytometry results further confirmed the excellent endocytosis of LZH nanozymes (Fig. S13). The fluorescence intensity of LZH was 6.0 times that of ZIF@HA (Fig. S14). Endocytosis was further characterized through hyperspectral imaging. As shown in Fig. S15, hyperspectral images of ZIF@HA and LZH NPs were collected to establish spectrum libraries. Then, the spectrum library was used to characterize ZIF@HA and LZH nanozymes in the cells. In the mapping images (Fig. 3e and f), the endocytosis of LZH nanozymes by cells was significantly greater than that of ZIF within the same time, which further proved that the flexibility of liquid metal can promote the endocytosis. Hence, the flexibility of liquid metal and the targeting of HA allow LZH nanozymes to be taken up by cells to a greater extent,thus greatly improving the efficacy of tumor MDT.

### Cell cytotoxicity in vitro and acute toxicity in vivo

**Fig. 4:**
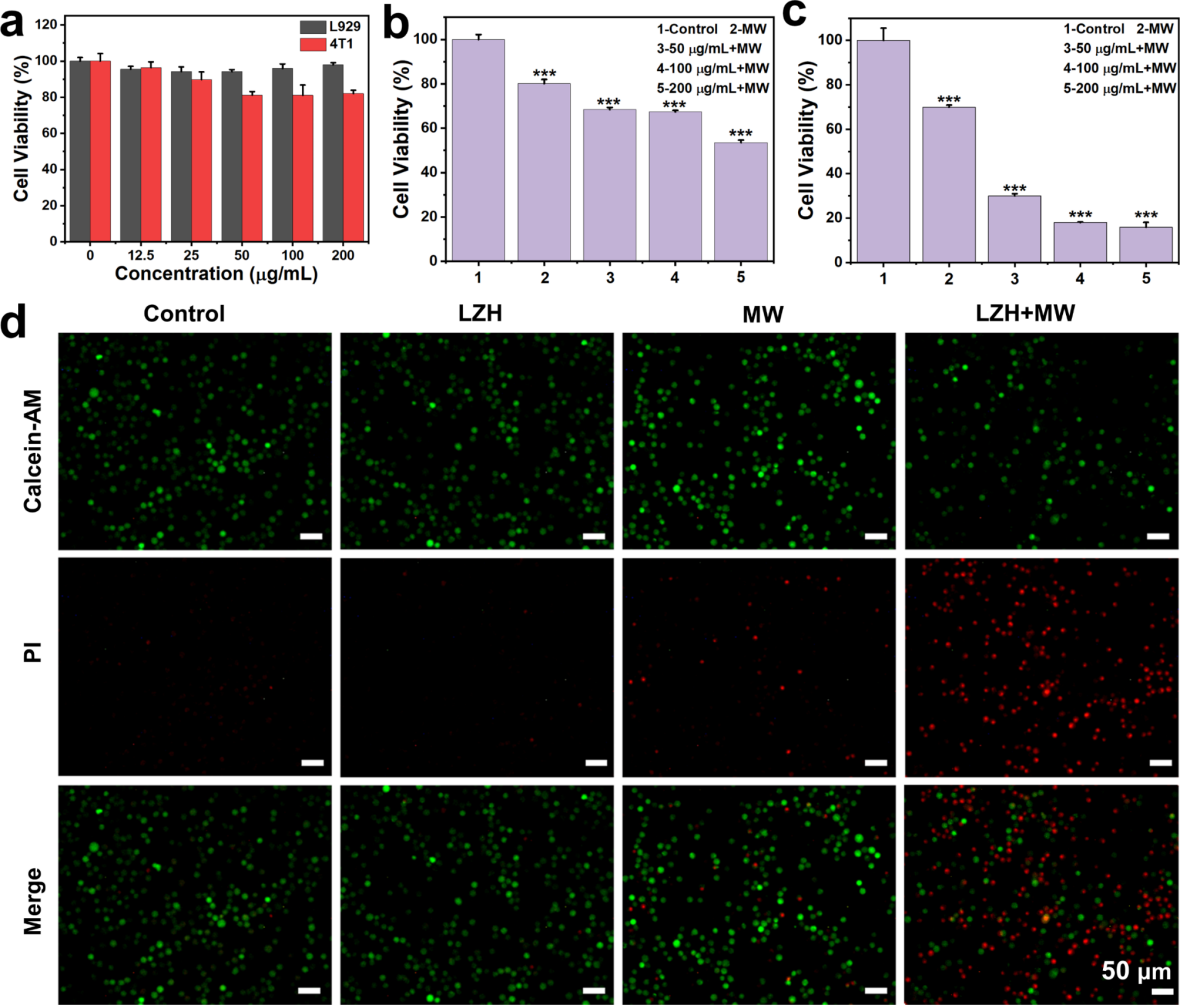
The cytotoxicity of LZH nanozymes and the inhibition of 4T1 cell growth. (**a**) The cytotoxicity of LZH nanozymes on L929 and 4T1 cells. (**b**) The inhibitory effect of LZH nanozymes on 4T1 cells under the MW at 0.9 W. (**c**) The inhibitory effect of LZH nanozymes on 4T1 cells under the MW at 1.8 W. (**d**) Fluorescence imaging of Calcein-AM and PI co-stained 4T1 cells under different treatment

Due to the excellent MW dynamic effect and oxygen self-supplying capacity, as well as targeting effect, LZH nanozymes are expected to be used in vivo to solve the defects of MDT. Therefore, the biosafety of LZH nanozymes needs to be evaluated. The cytotoxicity of LZH nanozymes was studied using mouse fibroblasts L929 and mouse breast cancer cells 4T1. The viability of 4T1 cells decreased slightly with the increase of LZH nanozymes concentration. At a high concentration of 200 µg/mL, the viability of L929 and 4T1 cells were 97 ± 1.1% and 82 ± 1.8% respectively (Fig. 4a), suggesting low toxicity of LZH nanozymes to cells. Besides, acute toxicity tests on LZH nanozymes were performed according to the ethical principles of animal experiments. The weight of the mice decreased slightly on the first day of treatment, but continued to rise over time (Fig. S16). H&E staining of major organs and blood routine analysis showed no obvious difference between the experimental group and the control group (Fig. S17 and S18), despite the injection concentration as high as 200 mg/kg. These results indicated that LZH nanozymes had excellent biological safety and could be used for tumor treatment in vitro and in vivo.

### Anticancer study in vitro

Before animal treatment experiments, we first assessed the inhibitory effect of LZH nanozymes at the cellular level. As shown in Fig. 4b and **c**, the viability of 4T1 cells in different treatment groups was measured respectively. Under the MW at 0.9 W, the cell viability of the MW group was about 80%, and when the power was increased to 1.8 W, the cell viability was reduced to about 69%. In addition, at the same power, as the concentration of LZH nanozymes increased, the cell viability also showed a relatively obvious downward trend. At the concentration of 100 µg/mL, the cell viability was only 18% under 1.8 W MW irradiation, which was extremely significant different from that of MW group, demonstrating that LZH nanozymes had a remarkable MDT in vitro at the cellular level. On this basis, a MW power of 1.8 W and a material concentration of 100 µg/mL were selected for live and dead staining of cells (Fig. 4d). LZH + MW group showed the weakest green fluorescence and the strongest red fluorescence, demonstrating that LZH nanozymes had the best inhibitory effect on tumor cells under MW irradiation. These proved the excellent MDT effect of LZH nanozymes once again.

### Computed tomography (CT) imaging

Studies show that the high density of liquid metal makes it promising to replace traditional iodine-based contrast agents and enhance the contrast of CT images [48]. Inspired by this, the CT imaging ability was also investigated before conducting in vivo therapy. As the concentration of LZ increased, the brightness gradually enhanced (Fig. S19a), manifesting that LZH nanozymes had CT imaging capability and is concentration-dependent. In vivo CT imaging was performed in the PDX model, the brightness of the tumor changed significantly before and after injection, and the average HU value of the tumor after injection was more than twice that before injection (Fig. S19b), demonstrating that LZH nanozymes could be used as a CT contrast agent for in vivo monitoring and treatment.

### Anticancer efficiency in vivo

**Fig. 5:**
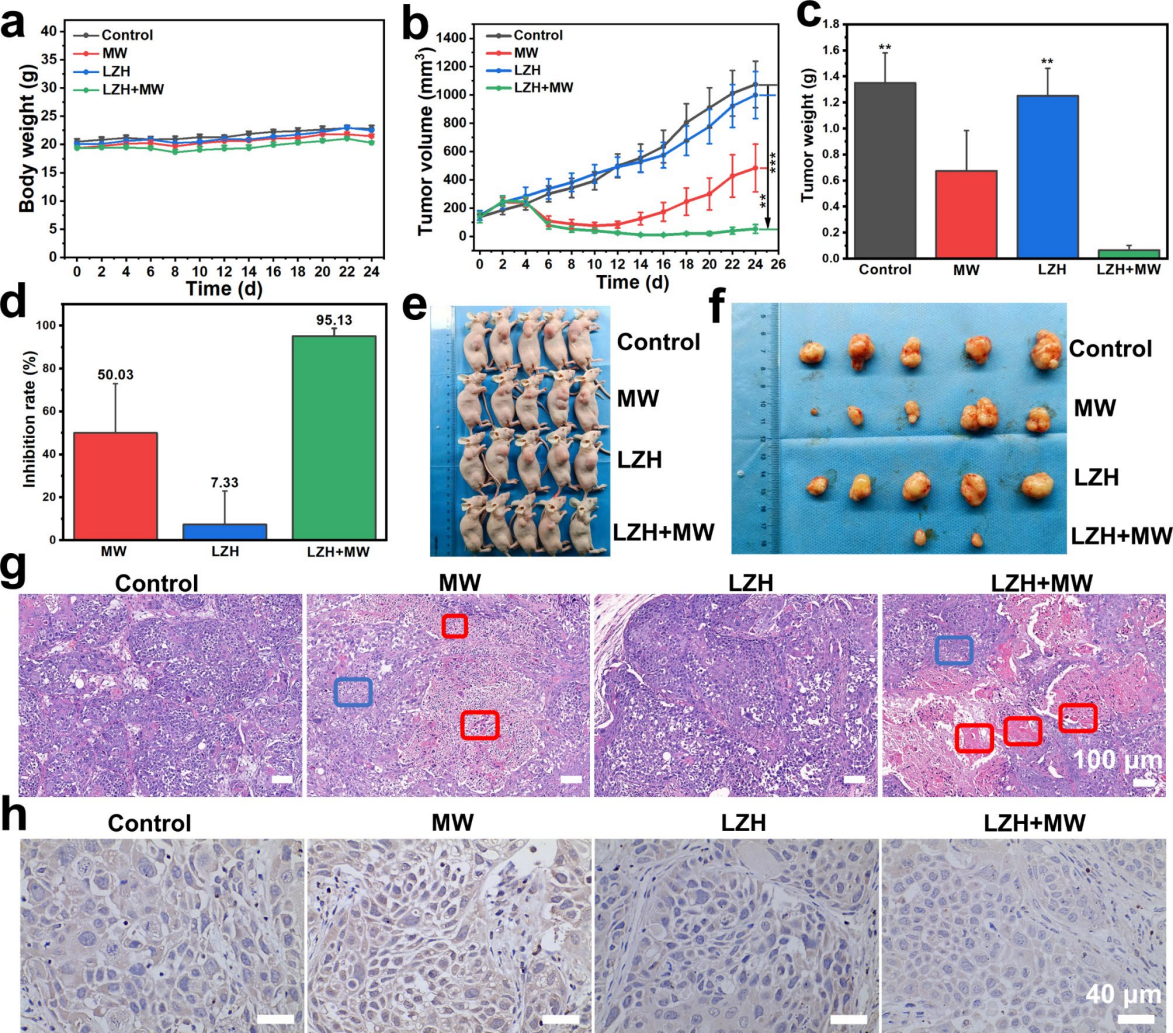
Anticancer in PDX model. (**a**) The body weight curve of mice during 24 days treatment. (**b**) The curve of tumor volume changes during 24 days. (**c**) Tumor weight of different groups. (**d**) Inhibition rate of different groups. (**e**) Photograph of tumor-bearing mice on the 24th day. (**f**) Photograph of tumors extracted from different groups. (**g**) H&E staining of tumor sections (red frame: necrotic cells; blue frame: living cells). (**h**) The expression of HIF-1α in different groups

**Fig. 6:**
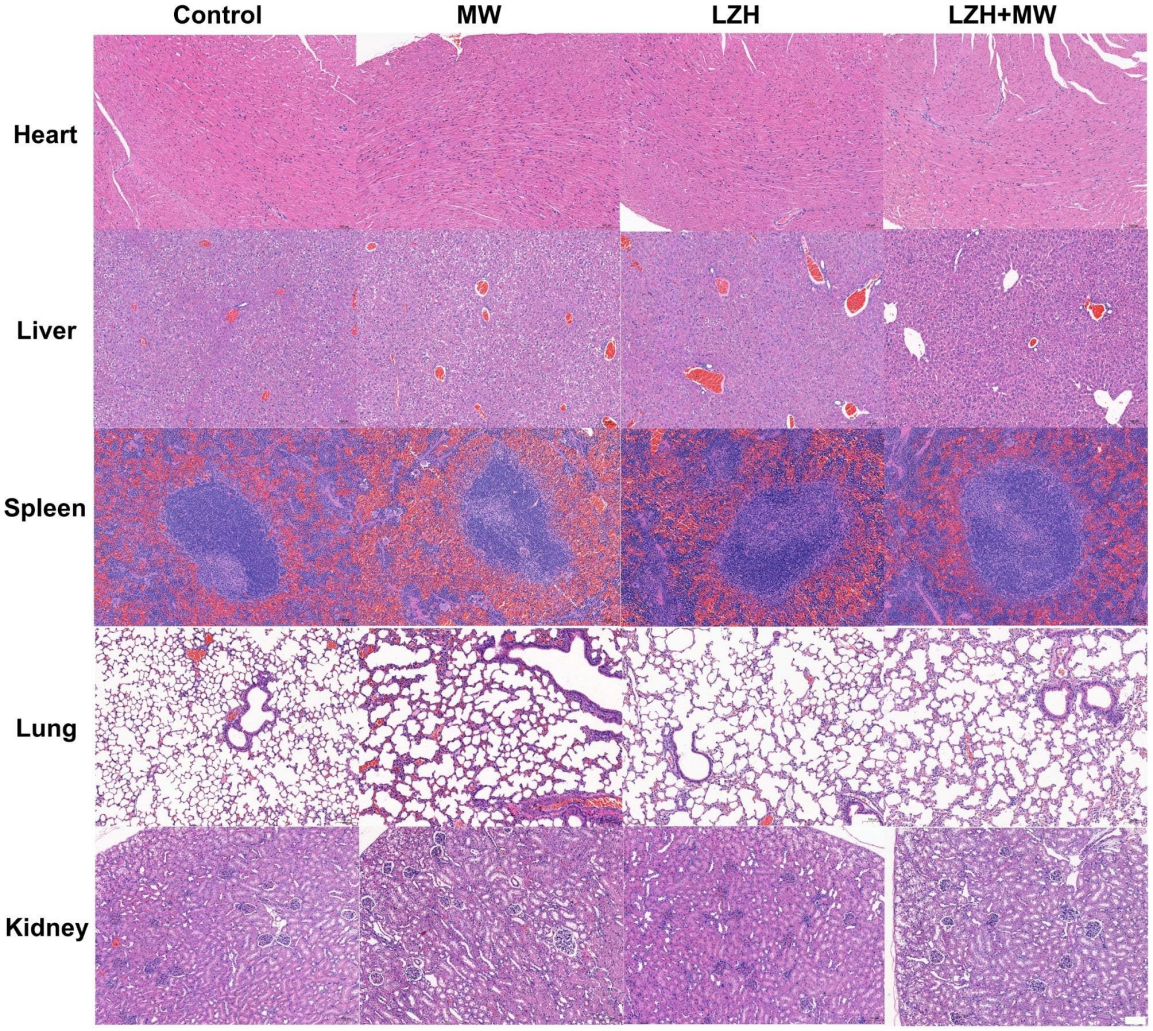
H&E staining of main organ sections of mice with different treatment (scale bar: 100 μm)

Compared with the model established by cancer cell lines cultured in vitro, PDX model can partially recreate the tumor hypoxic microenvironment and maintain similar histological and pathological features to the patients [41–43, 49]. For the purpose of retaining the heterogeneity of varying degrees of complexity found in human cancers, PDX model mice of breast cancer were established and randomly divided into 4 groups: Control, MW, LZH, LZH + MW. The body weight of all groups was no obvious difference compared with the control group during 24 days after treatment (Fig. 5a). Tumor volume of mice in the control group and the LZH group increased sharply, while that in the MW group and the LZH + MW group increased first and then gradually reduced (Fig. 5b**)**. This might be because the growth of tumors was faster than necrosis initially after MW irradiation, then the part of the necrosis was more than that of the growth. From the 6th day, the tumor volume in MW group began to increase, which may be due to incomplete tumor necrosis caused by simple MW treatment, leading to tumor recurrence. In contrast, the tumor volume in LZH + MW group was gradually decreased due to the effect of ROS on tumors. After 24 days, tumor volume in LZH + MW group was about 1/20 of that in control group, three mice were even completely cured. The tumor inhibition rate was as high as 95.13% (only 50% in MW group) (Fig. 5c and **d**), confirming that LZH nanozymes had excellent anti-tumor effect under MW irradiation. This was also verified by photograph of tumor tissue and mice (Fig. 5e and **f**). Figure 5 g exhibited the most cell necrosis in the LZH + MW group. The expression of HIF-1α in tumor tissues under different treatments was characterized by immunohistochemistry (Fig. 5h). It can be seen that the expression of HIF-1α in LZH group was lower, indicating that LZH nanozymes can alleviate tumor hypoxia to a large extent. Under MW irradiation, the degree of hypoxia was further relieved. The immunohistochemical intensity of the LZH + MW group was 0.4 times that of the control group (Fig. S20), 0.3 times that of the MW group, and 0.8 times that of the LZH group. This proved that LZH nanozymes still had excellent oxygen production ability in vivo, and could effectively improve tumor oxygen deficiency. Additionally, H&E staining of tissue sections also revealed that LZH nanozymes had no obvious toxicity to major organs (Fig. 6), indicating that LZH nanozymes had good biocompatibility. These results manifested that LZH nanozymes with low toxicity had excellent therapeutic effects of MDT.

## Conclusion

In conclusion, we first successfully achieved the binding of liquid metal and ZIF by simple electrostatic adsorption, and obtained a liquid metal-based nanozyme LZH with good stability through HA coating. This synthetic LZH nanozyme had certain flexibility derived from liquid metal, which could effectively promote the cell internalization of LZH nanozyme. HA endowed it with targeting ability, further promoted the endocytosis of the material, and doubly solved the problem of poor delivery of microwave sensitizers. Due to the CAT-like activity, endogenous H_2_O_2_ in TME was catalyzed by LZH nanozyme to produce O_2_. The increase of O_2_ level could greatly enhance the production of ROS, efficiently eliminate the restriction of MDT, and further induce the apoptosis and death of residual tumor cells. Cell experiments showed that LZH nanozyme had an excellent inhibitory effect on tumor cells. In addition, the PDX model of breast cancer was established to evaluate the therapeutic effect of LZH-mediated MDT. The in vivo studies demonstrated that LZH nanozyme had a significant tumor therapeutic effect under MW irradiation, and the tumor inhibition rate reached 95%. The successful synthesis of LZH nanozyme provides simple strategy for the surface modification of liquid metal, while its excellent MDT in PDX model promises broad application prospects in the treatment of clinical breast cancer.

### Electronic supplementary material

Below is the link to the electronic supplementary material.

Supplementary Material 1

## Data Availability

The datasets used and/or analysed during the current study are available from the corresponding author on reasonable request.
