# Supplementary material for: Engineering liquid metal-based nanozyme for enhancing microwave dynamic therapy in breast cancer PDX model
Source: J Nanobiotechnology. 2023 Oct 31;21:399. doi: 10.1186/s12951-023-02121-9 (PMC10617232; doi:10.1186/s12951-023-02121-9)
Supplement: Supplementary file 1 — Supplementary Material 1 [file 12951_2023_2121_MOESM1_ESM.docx]

**Engineering Liquid Metal-Based Nanozyme for Enhancing Microwave Dynamic Therapy in Breast Cancer PDX Model**

Qiong Wu^1^^,5#^, Yongnian Yu^1,2,#^, Xiaorui Yu^2^, Qijun Du^3,4^, Li Gou^2,^*, Longfei Tan^1,5^, Changhui Fu^1,5^, Xiangling Ren^1,5^, Jun Ren^1,5^, Kai Xiao^4,^*, Xianwei Meng^1,5,^*

^1^ Laboratory of Controllable Preparation and Application of Nanomaterials, Technical Institute of Physics and Chemistry, Chinese Academy of Sciences, Beijing 100190, China

^2^ College of Biomedical Engineering, Sichuan University, Chengdu 610065 China

^3^ Sichuan Kangcheng Biotechnology Co., LTD, No.28 Gaopeng Avenue, High-tech Zone, Chengdu, 610000, China

^4^ Precision Medicine Research Center & Sichuan Provincial Key Laboratory of Precision Medicine and National Clinical Research Center for Geriatrics, West China Hospital, Sichuan University, Chengdu 610041, China

^5^ CAS Key Laboratory of Cryogenics, Technical Institute of Physics and Chemistry, Beijing 100190, China

^*^ Corresponding author. E-mail addresses: mengxw@mail.ipc.ac.cn, gouli@scu.edu.cn, xiaokaikaixiao@scu.edu.cn.

*
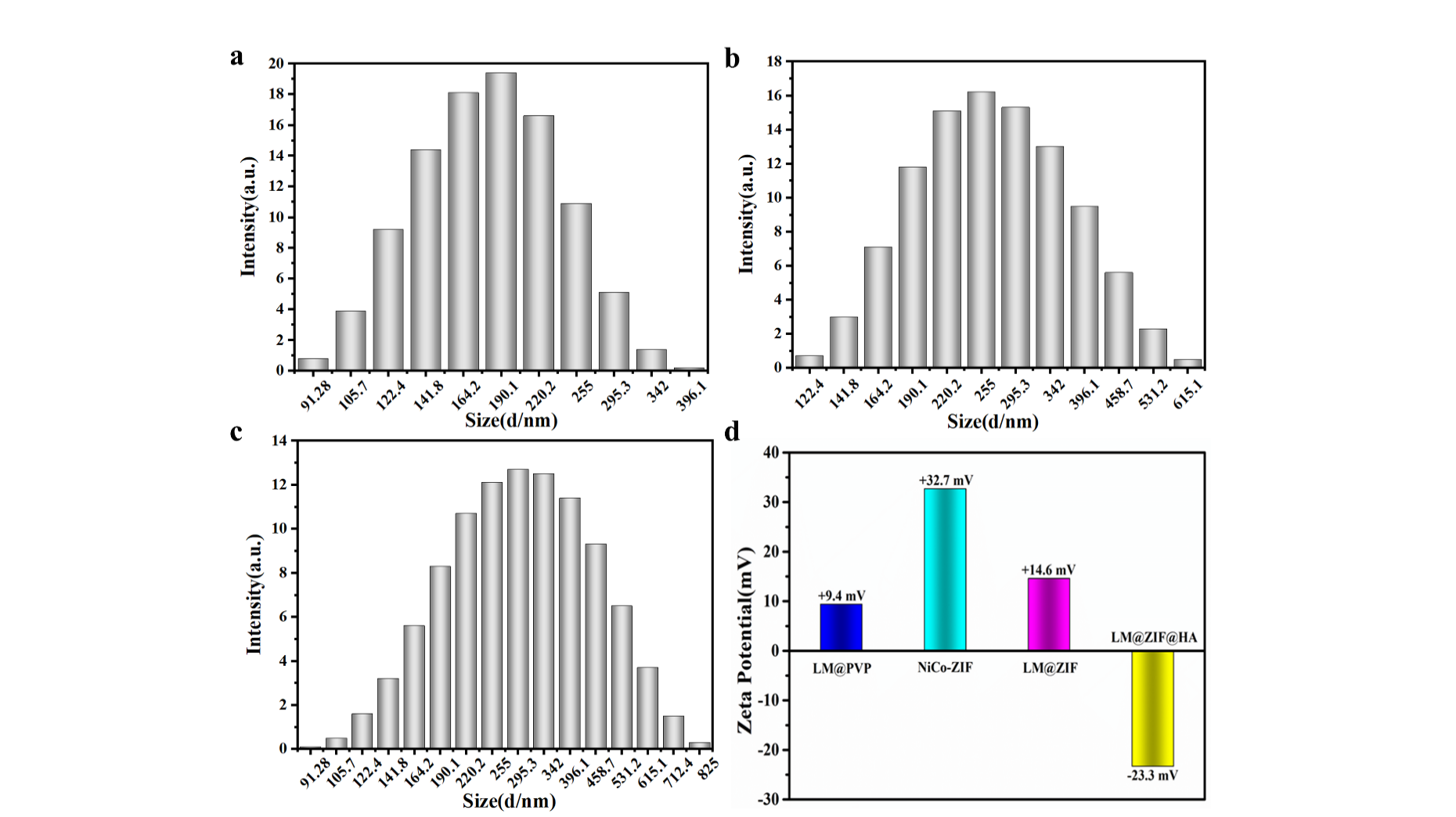
*

**Fig. S1** Hydrodynamic size and Zeta potential of different materials. a) Hydrated particle size of LM@PVP NPs. b) Hydrated particle size of LZ NPs. c) Hydrated particle size of LZH nanozymes. d) Zeta potential of different materials.


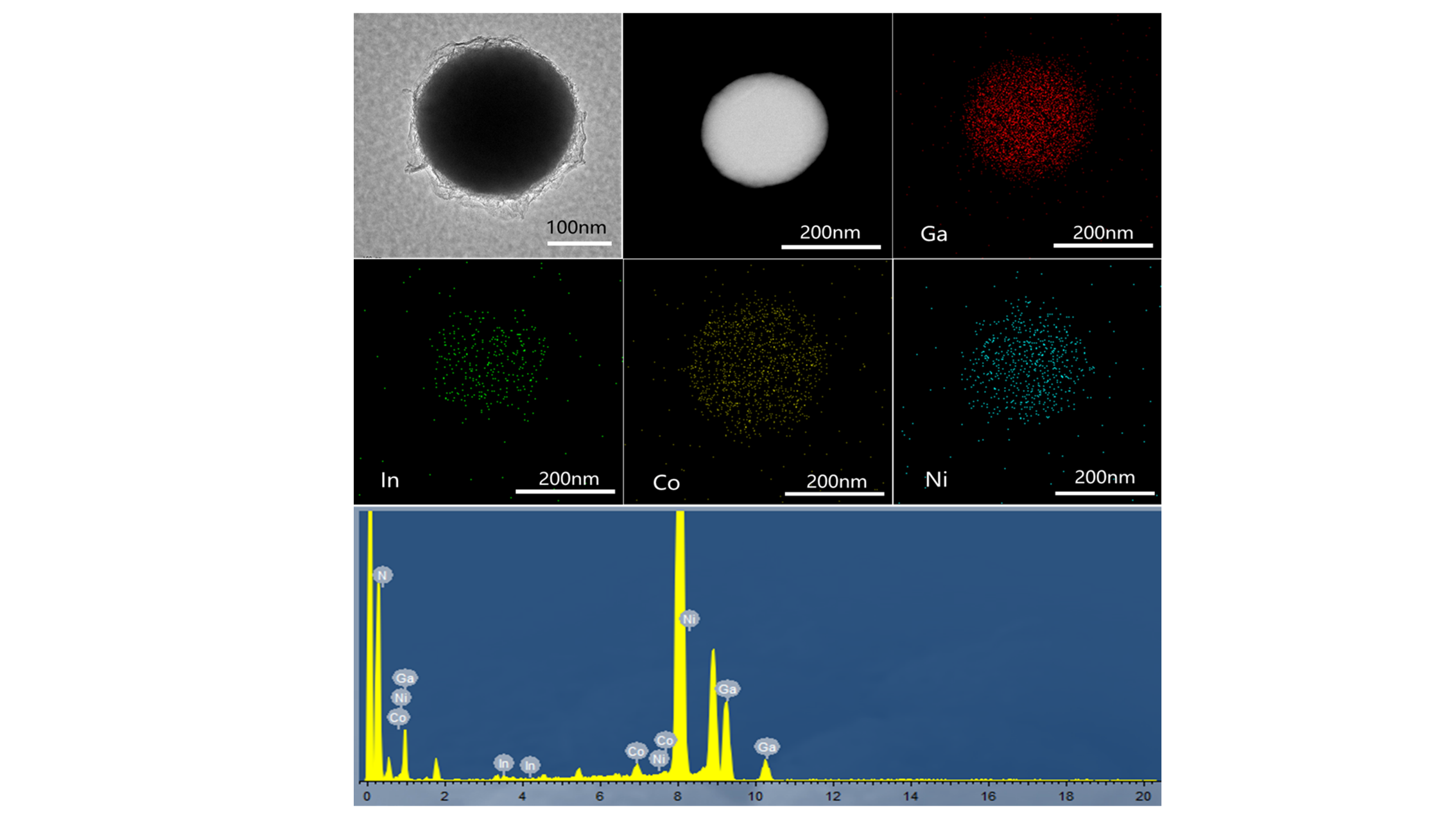


**Fig. S2** EDS element mapping images of LZH nanozymes and corresponding EDS.


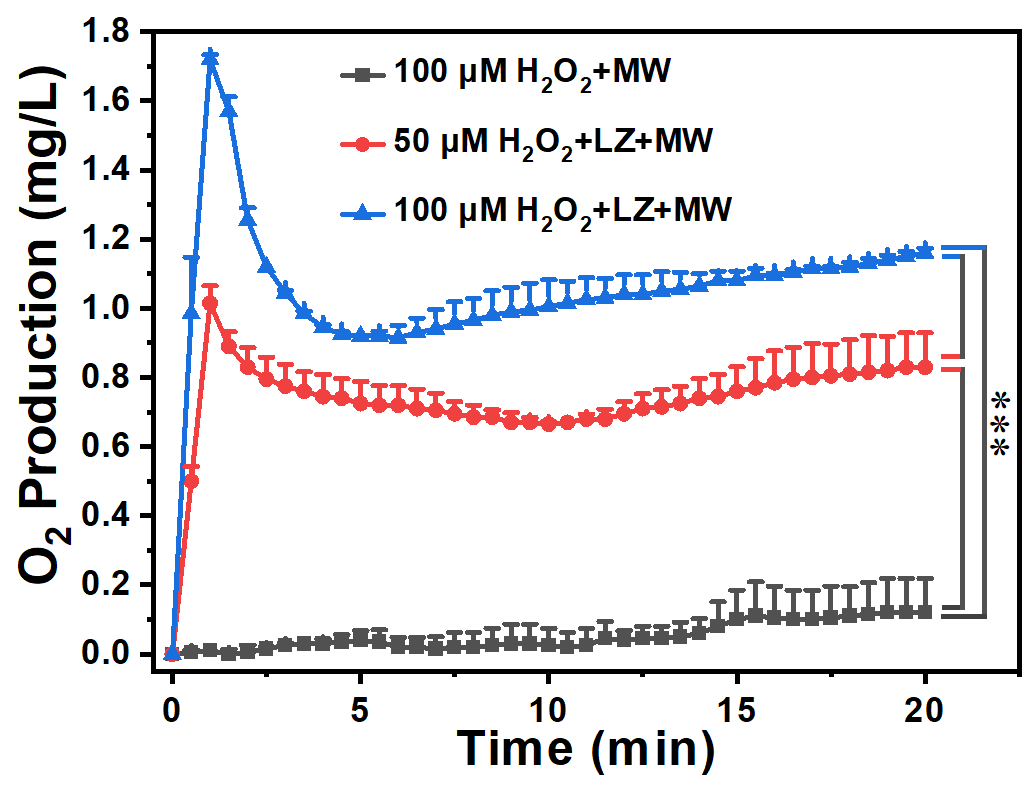


**Fig. S3** The CAT-like activity of LZ NPs under microwave irradiation at different H_2_O_2_ concentrations (50 and 100 μM).


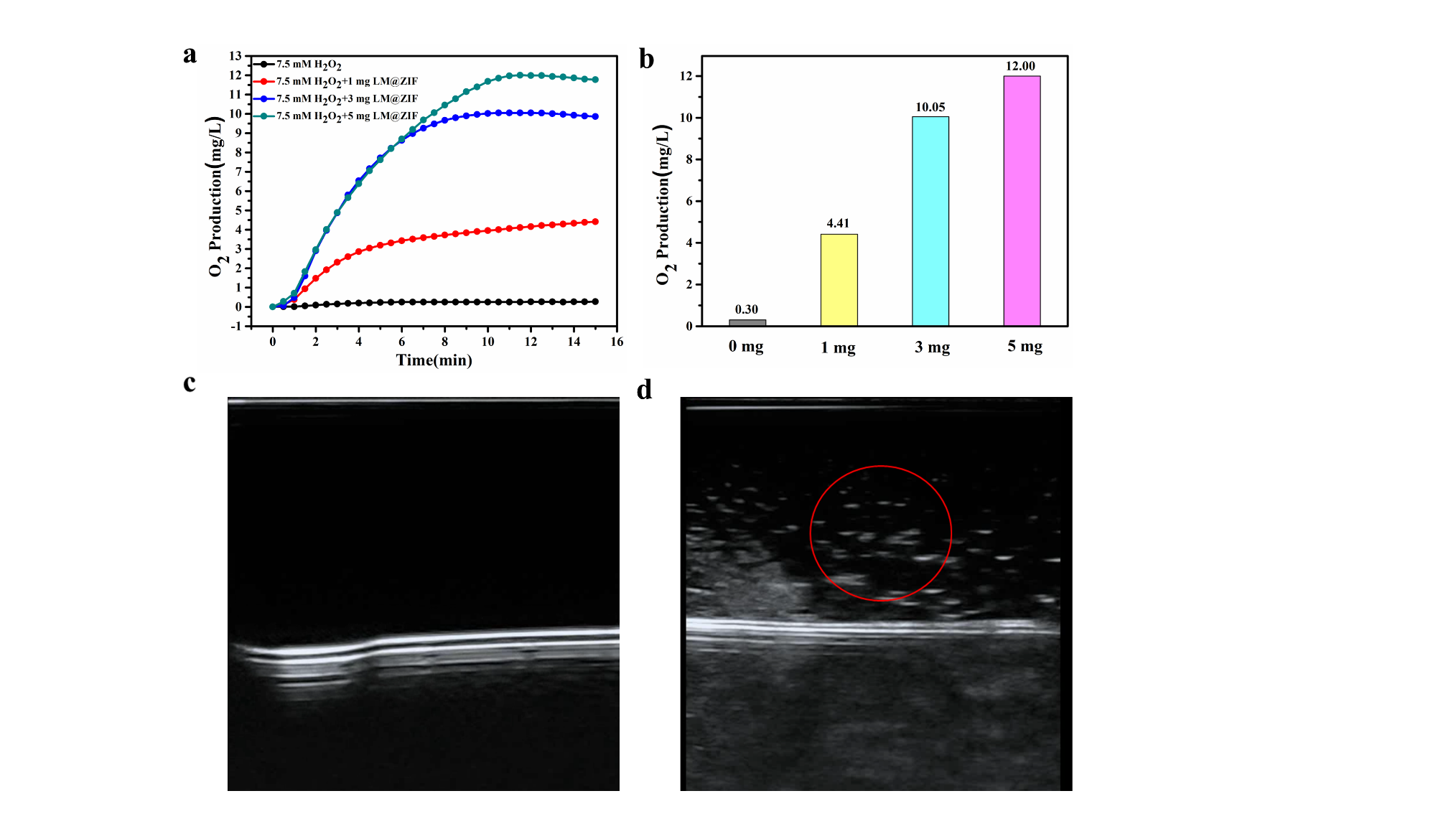


**Fig. S4** Characterization of oxygen generation. a) Oxygen production diagrams of different concentrations of LZ NPs (0, 1, 3, 5 mg/mL). b) The net difference of oxygen production. c) B-scan ultrasonography of H_2_O_2_. d) B-scan ultrasonography of LZ+H_2_O_2_ (the red circle represents partial oxygen bubbles).


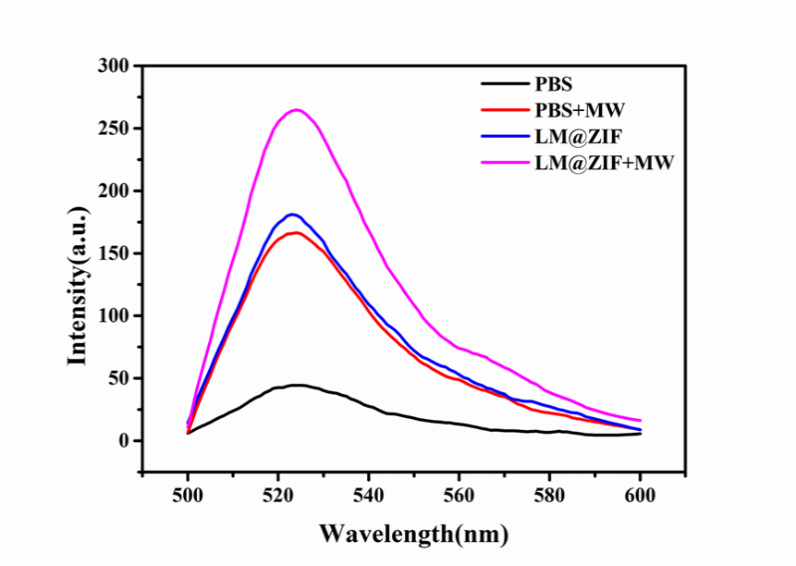


**Fig. S5** Fluorescence intensity of DCF under different treatments.


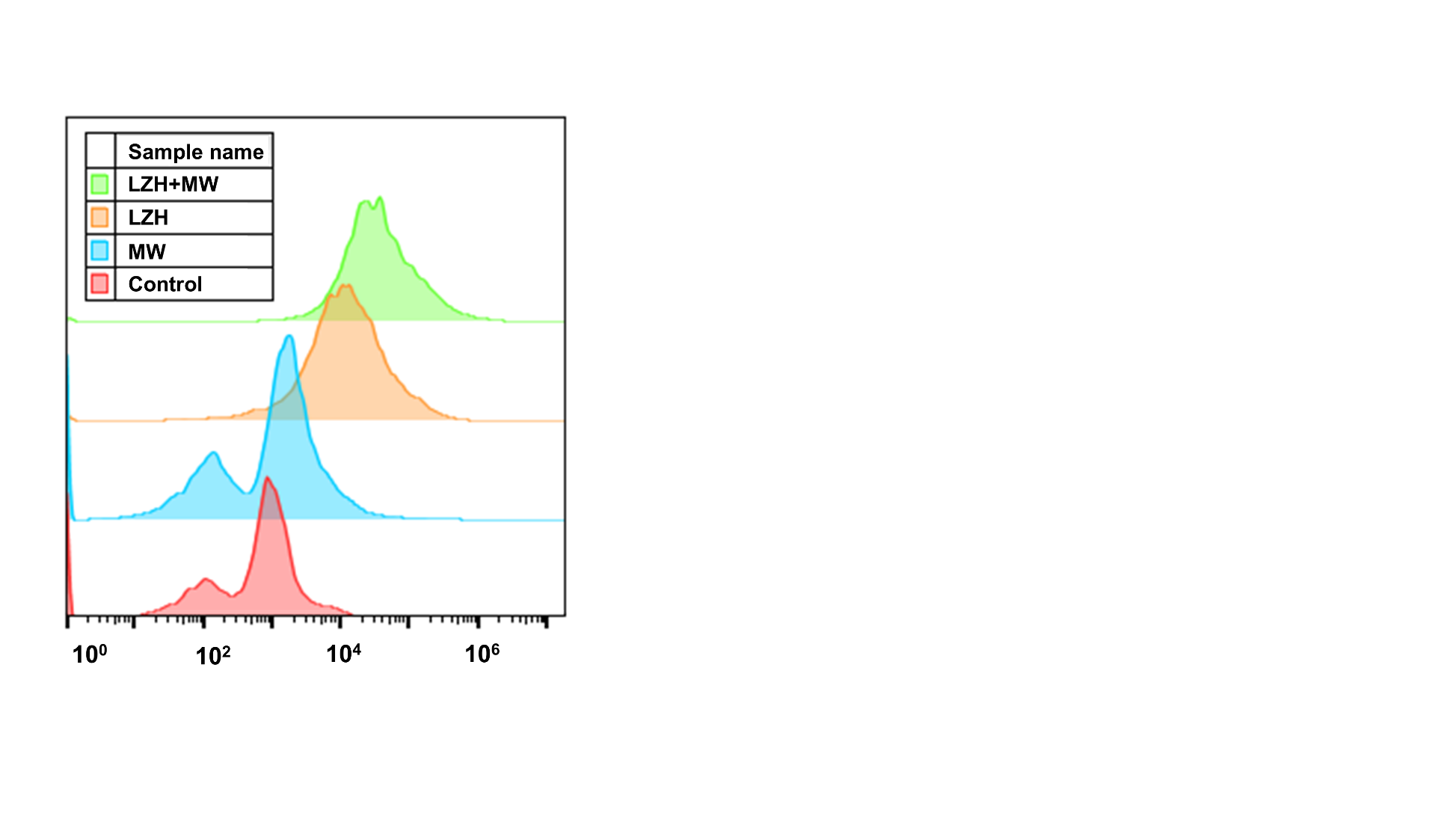


**Fig. S6** Flow cytometry results of ROS production at cellular level.


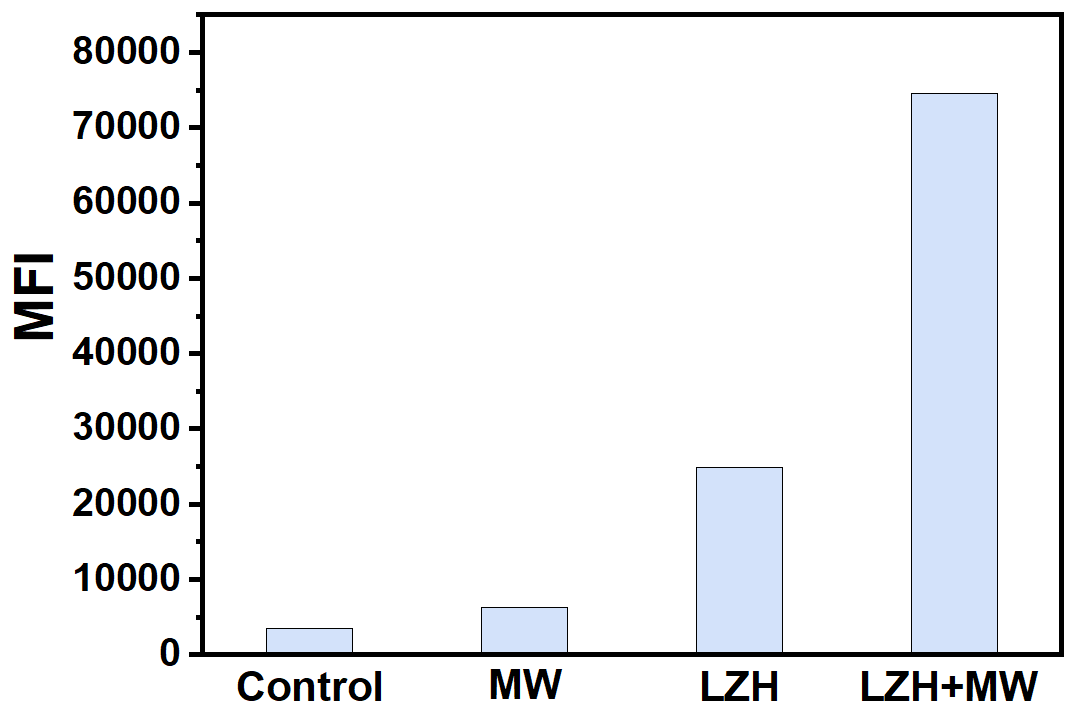


**Fig. S7** Quantitative flow cytometric analysis of ROS production at cellular level.


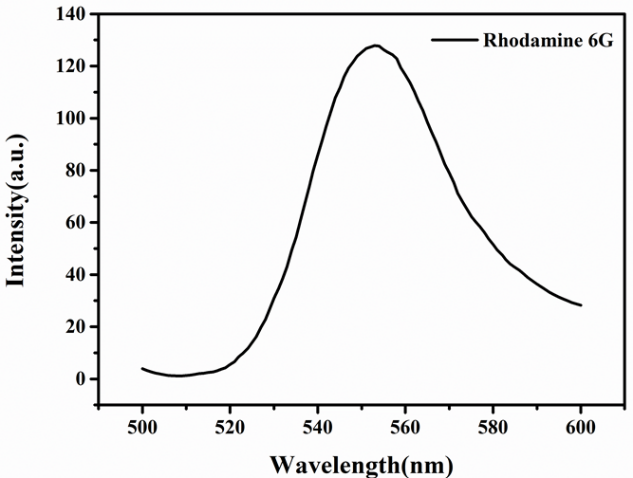


**Fig. S8** Fluorescence spectra of Rhodamine 6G.

**
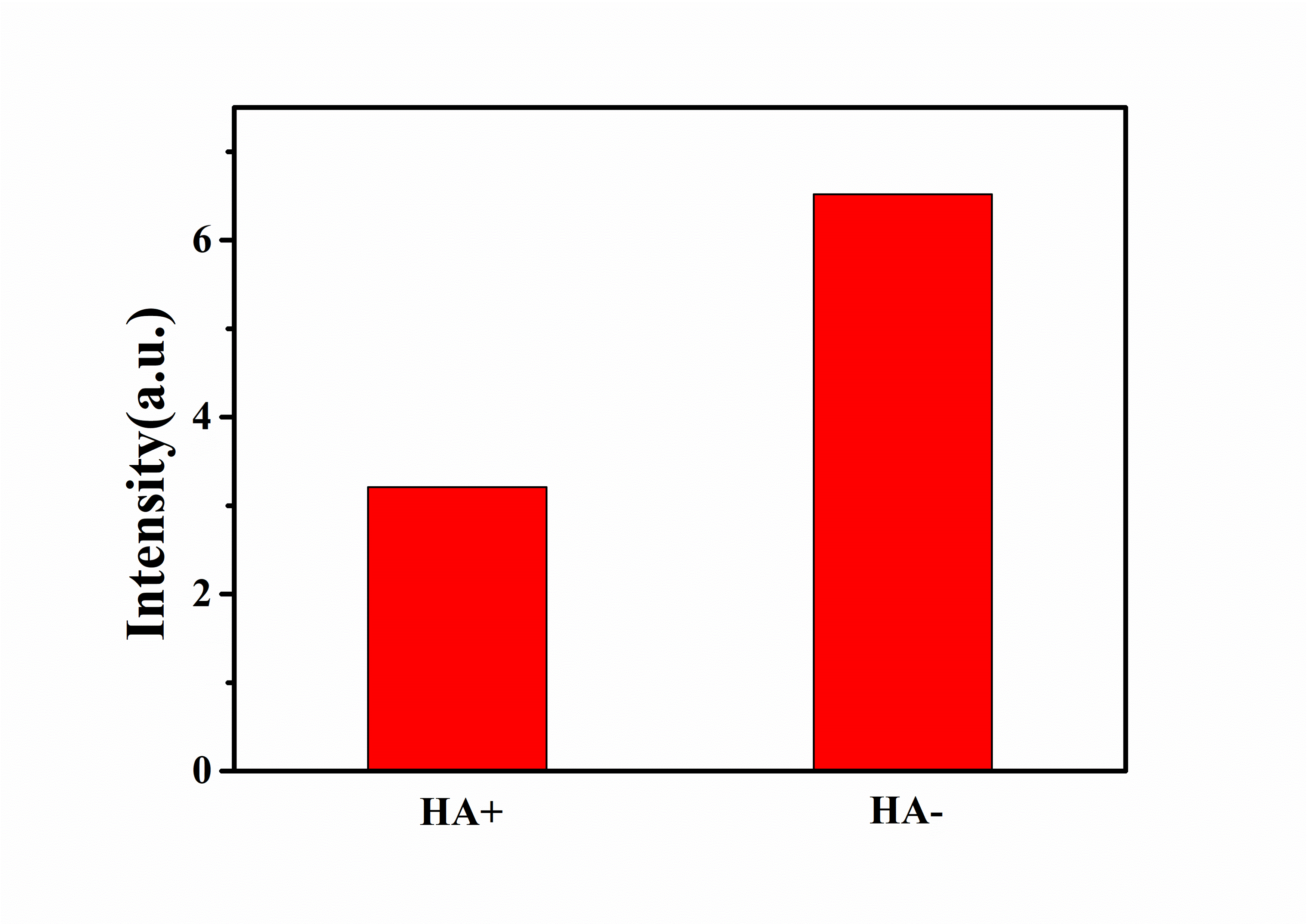
**

**Fig. S9** Mean fluorescence intensity of Rhodamine 6G in targeting performance of HA.


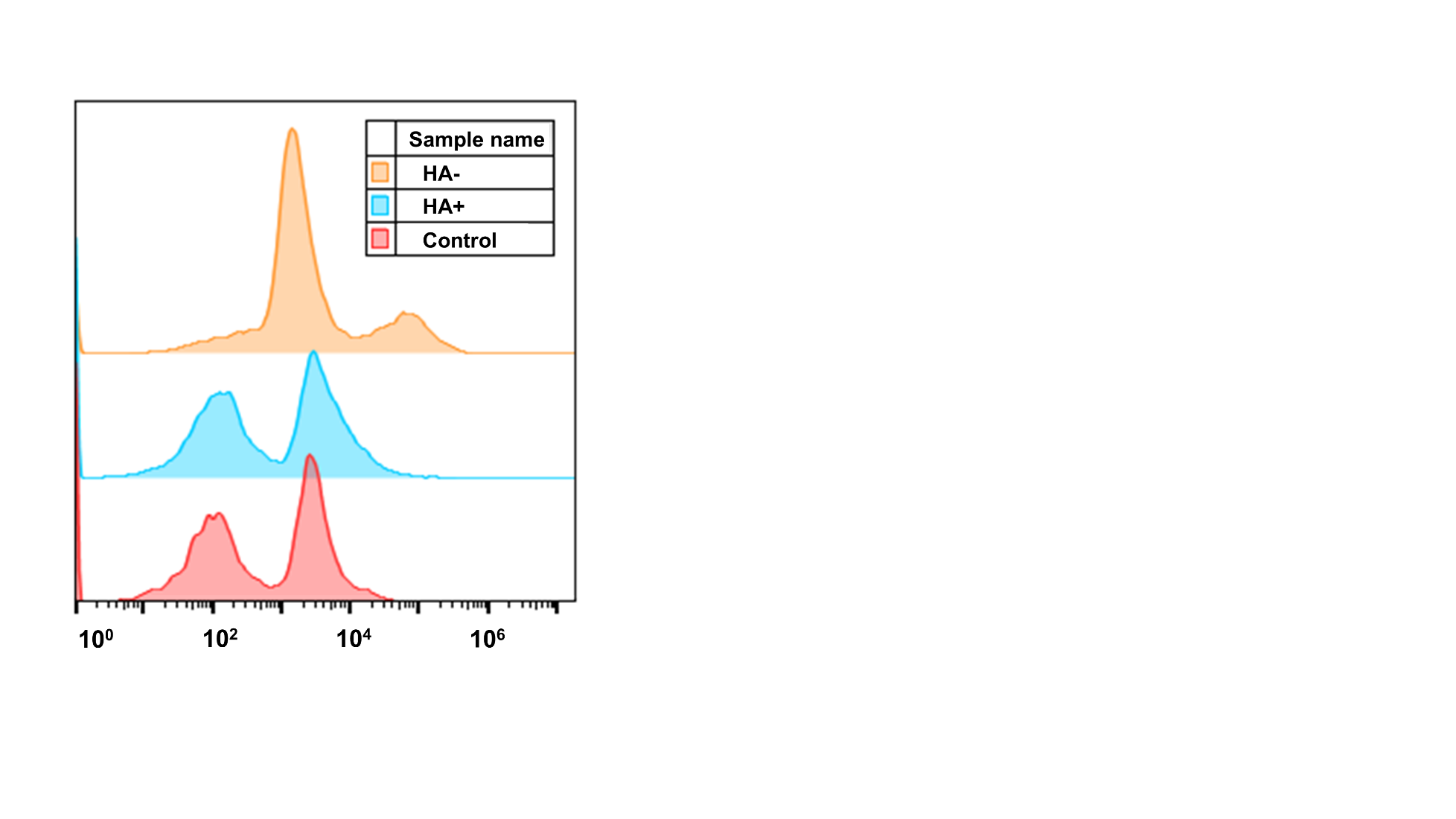


**Fig. S10** Flow cytometry results of CD44 targeting performance.


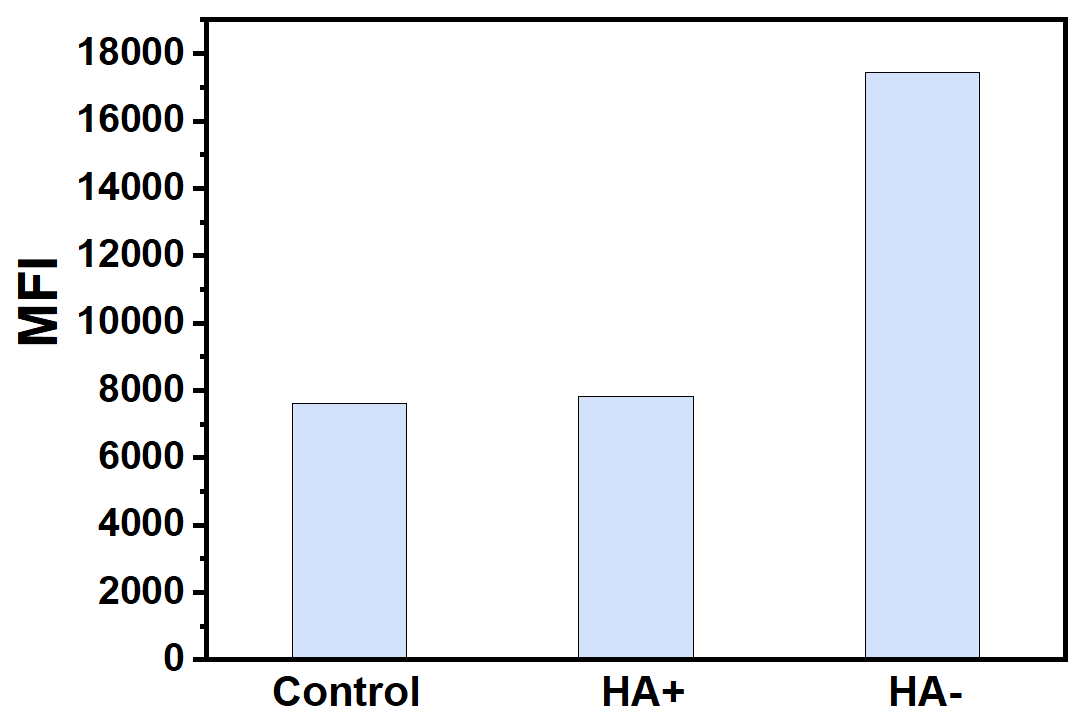


**Fig. S11** Quantitative flow cytometric analysis of CD44 targeting performance.


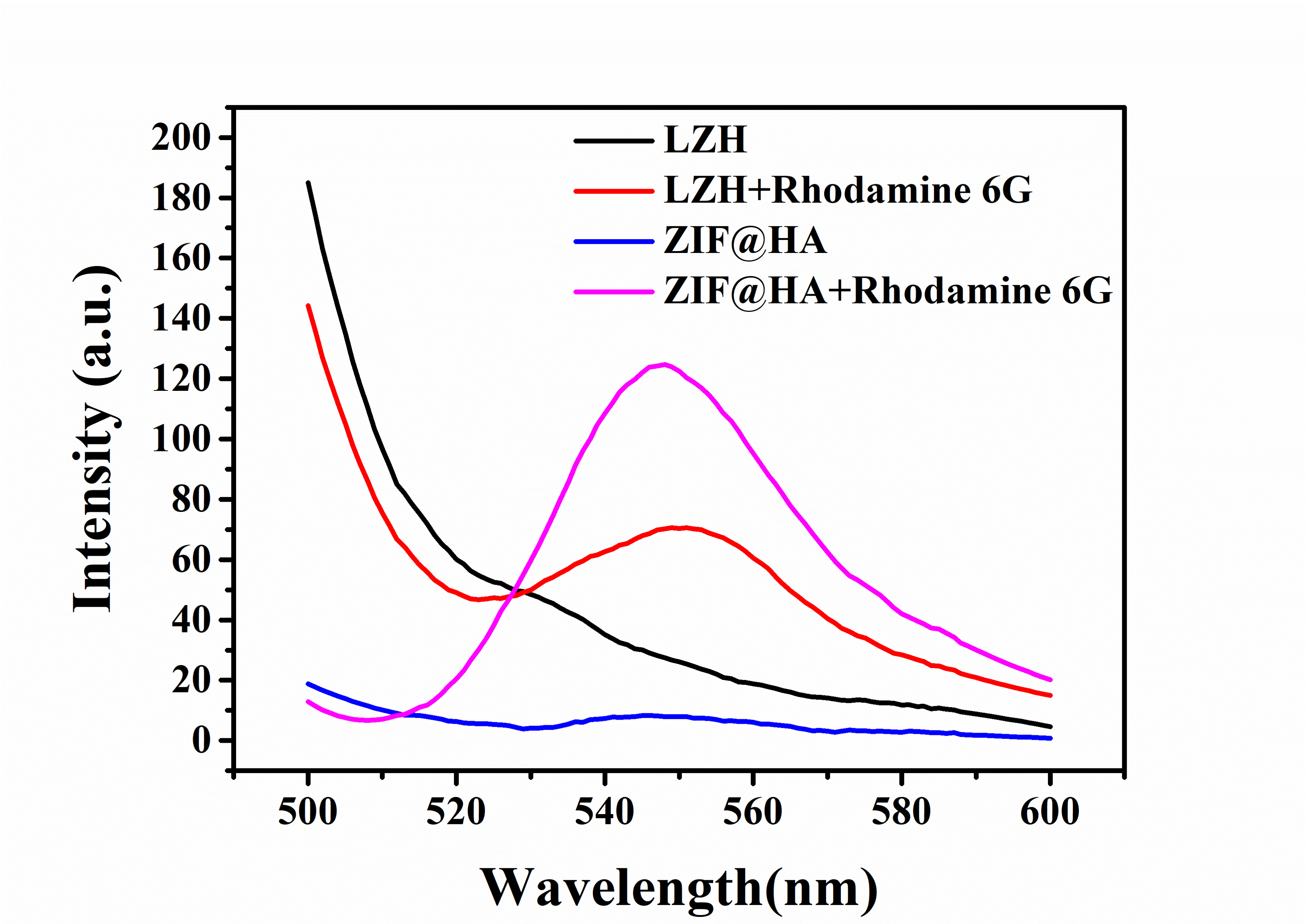


**Fig. S12** Fluorescence absorption of ZIF@HA and LZH nanozymes loaded with Rhodamine 6G.


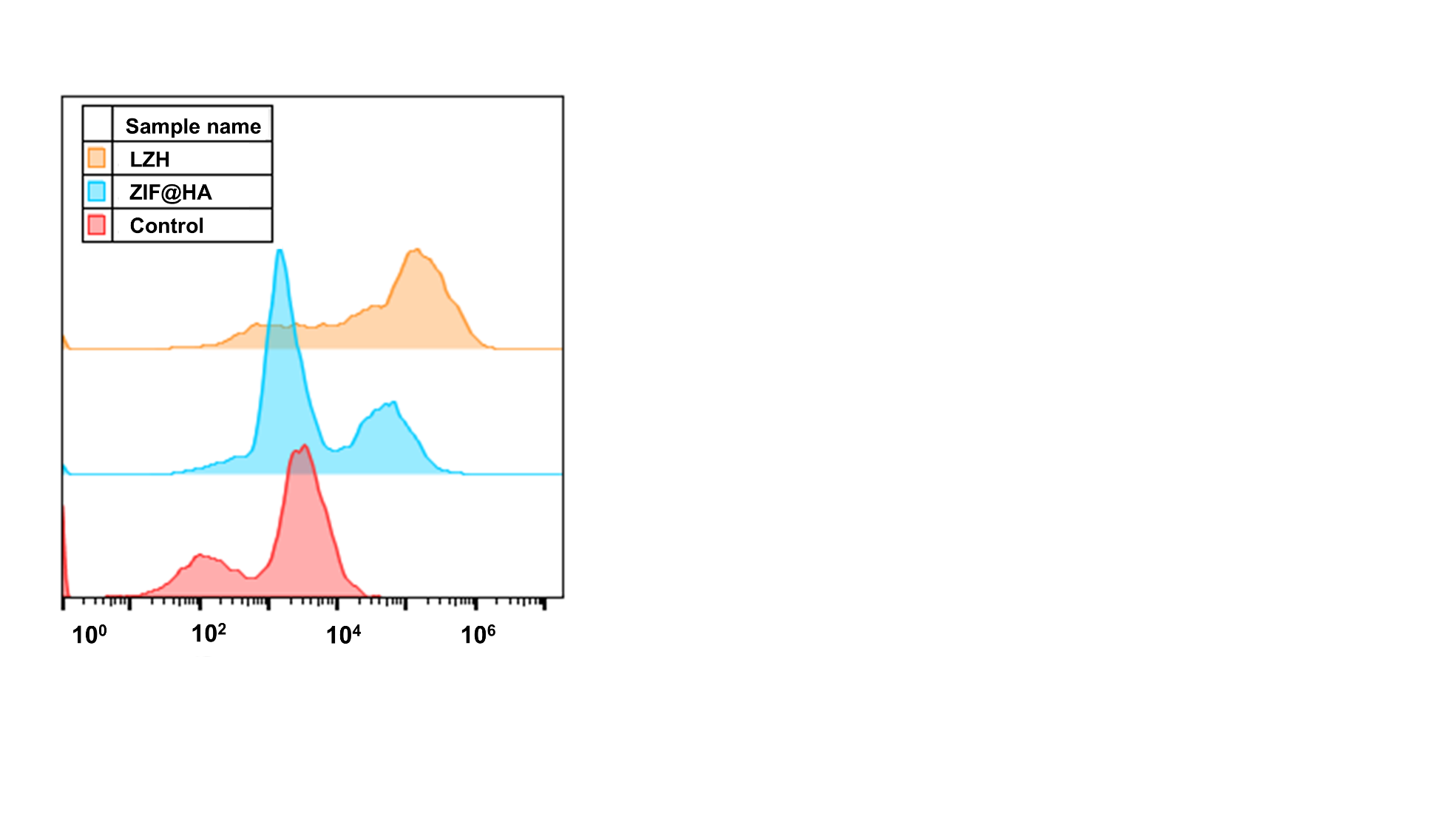


**Fig. S13** Flow cytometry results of cellular endocytosis.


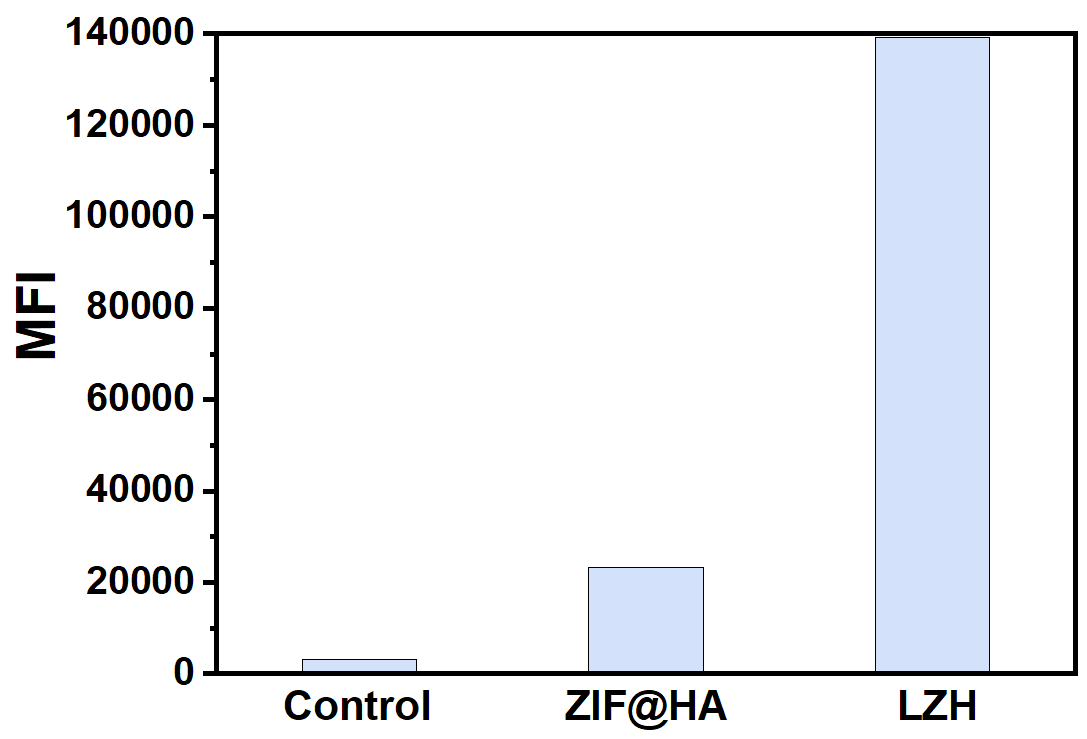


**Fig. S14** Quantitative flow cytometric analysis of cellular endocytosis.


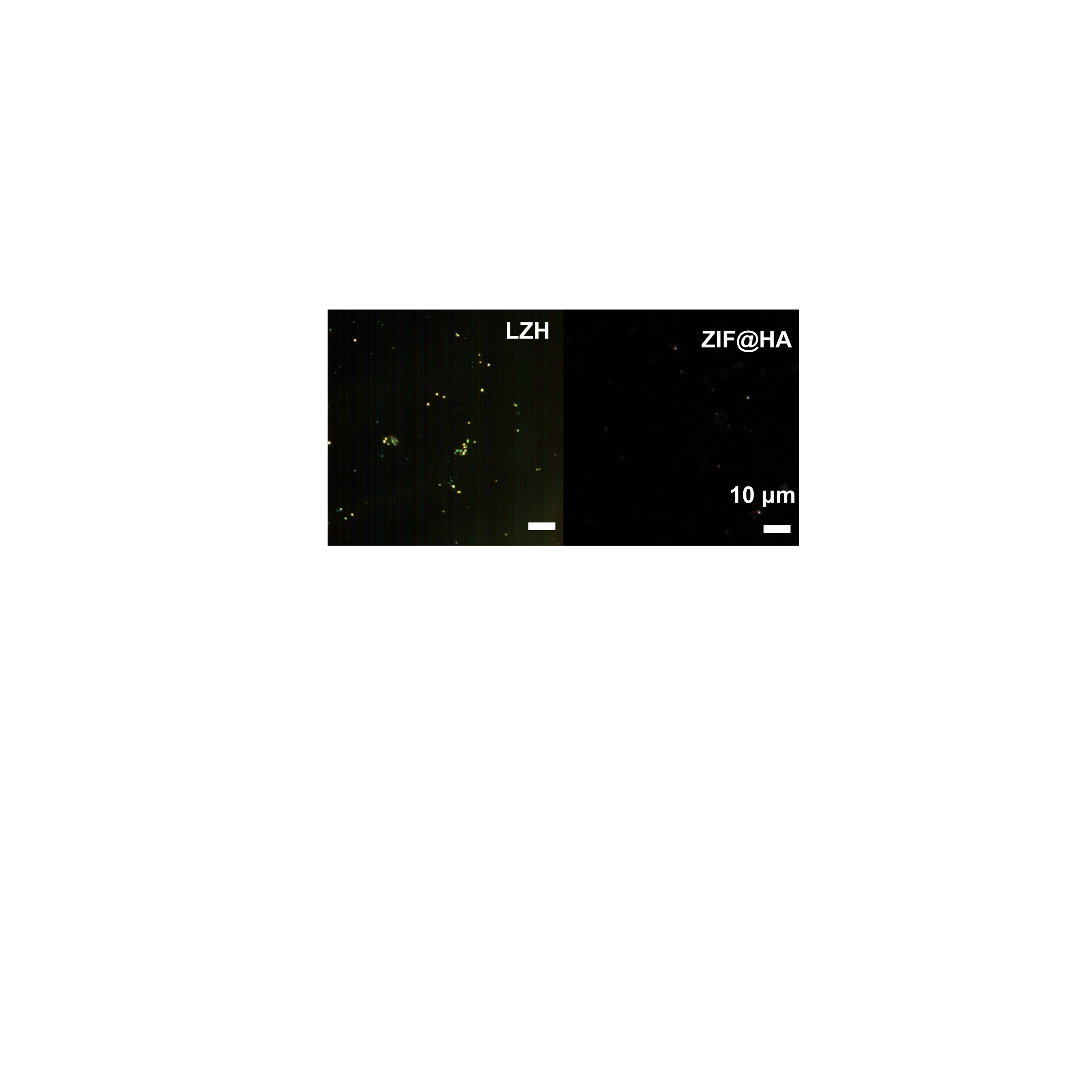


**Fig. S15** Hyperspectral images of ZIF@HA and LZH nanozymes.


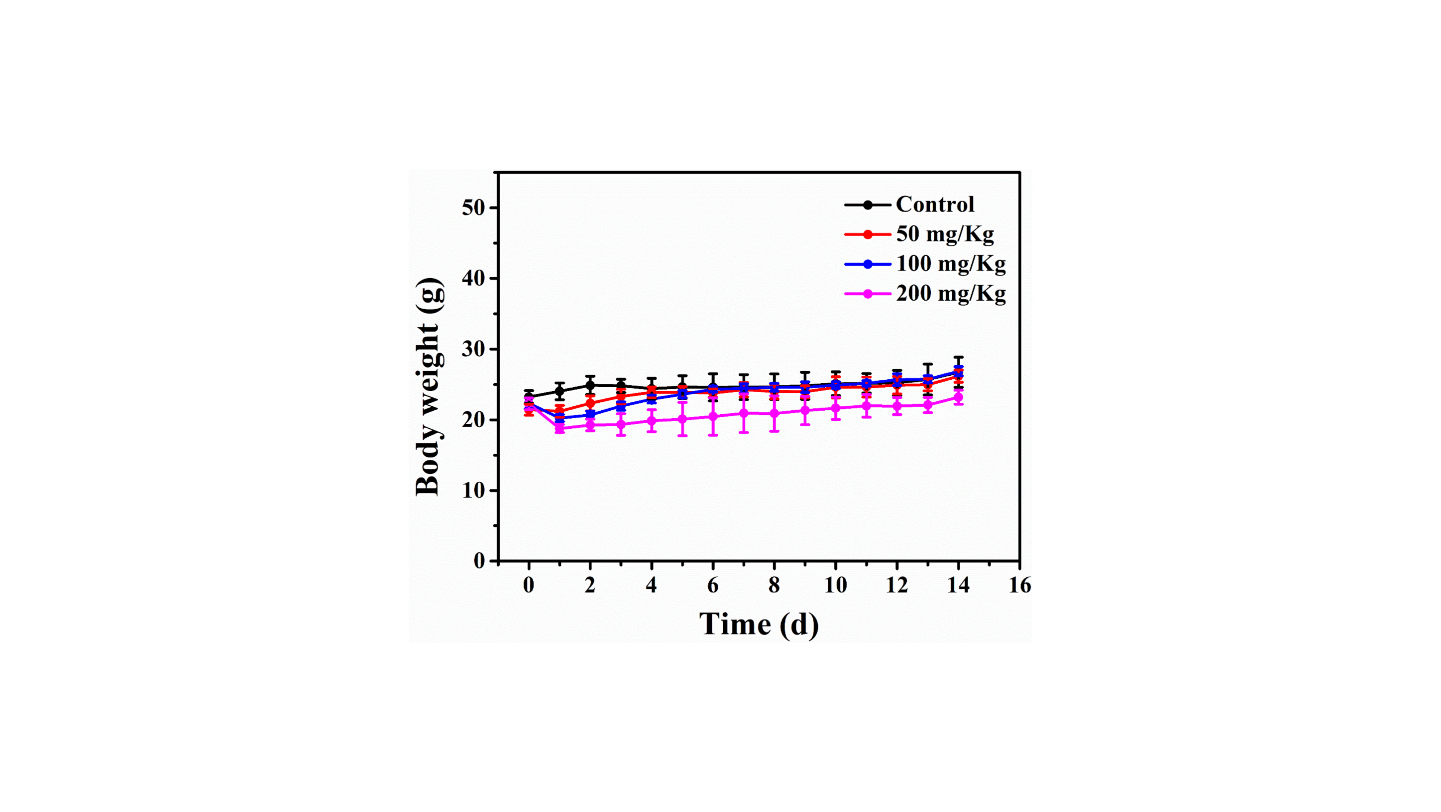


**Fig. S16** The weight change curve of the mice.


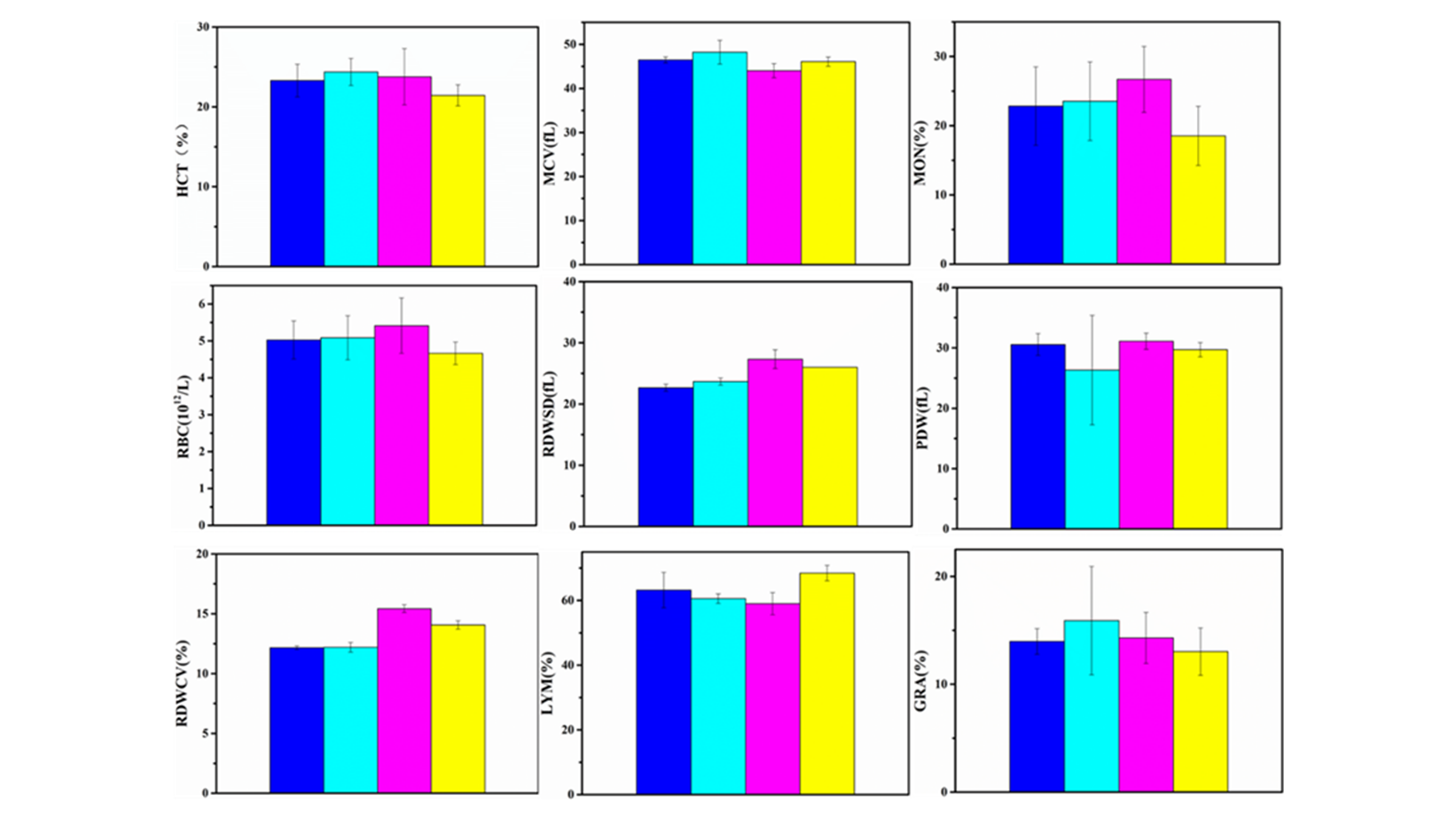


**Fig. S17** Blood routine analysis (blue: 0 mg/kg, cyan: 50 mg/kg, magenta: 100 mg/kg, yellow: 200 mg/kg).


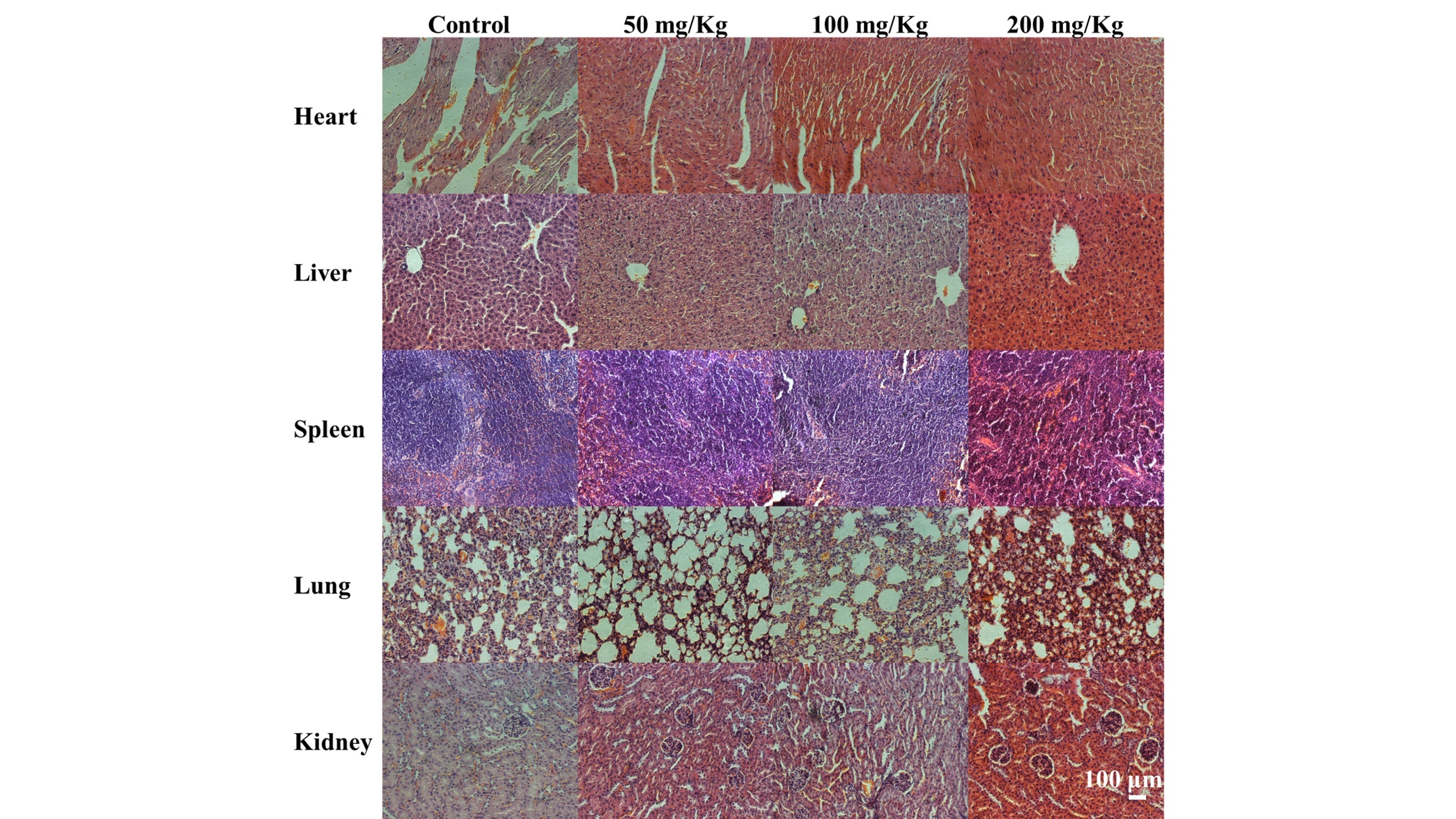


**Fig. S18** H&E staining of the main organ sections of the mice.


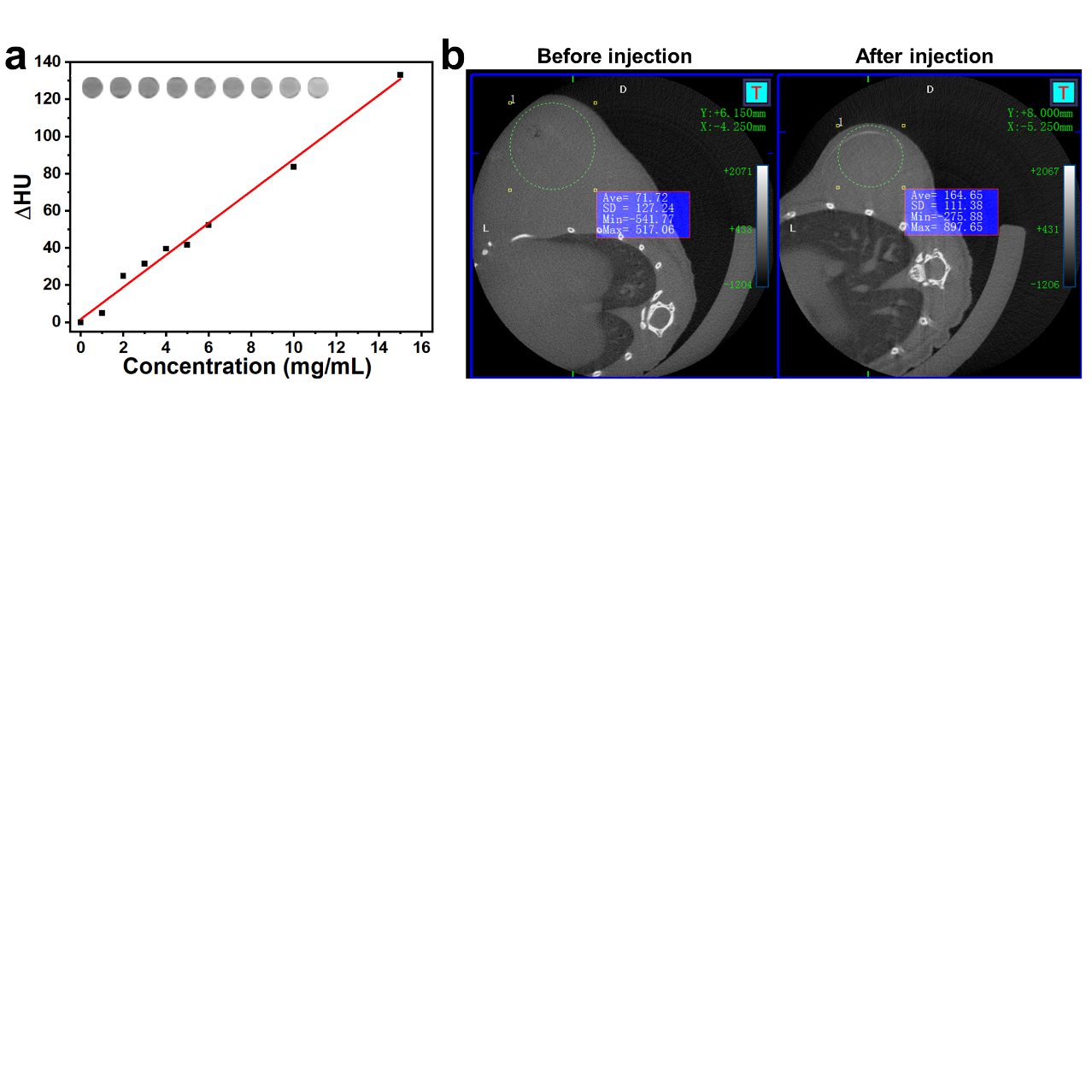


**Fig. S19** CT imaging. a) CT imaging and HU values of LZ NPs. b) CT imaging in vivo before injection and after injection.


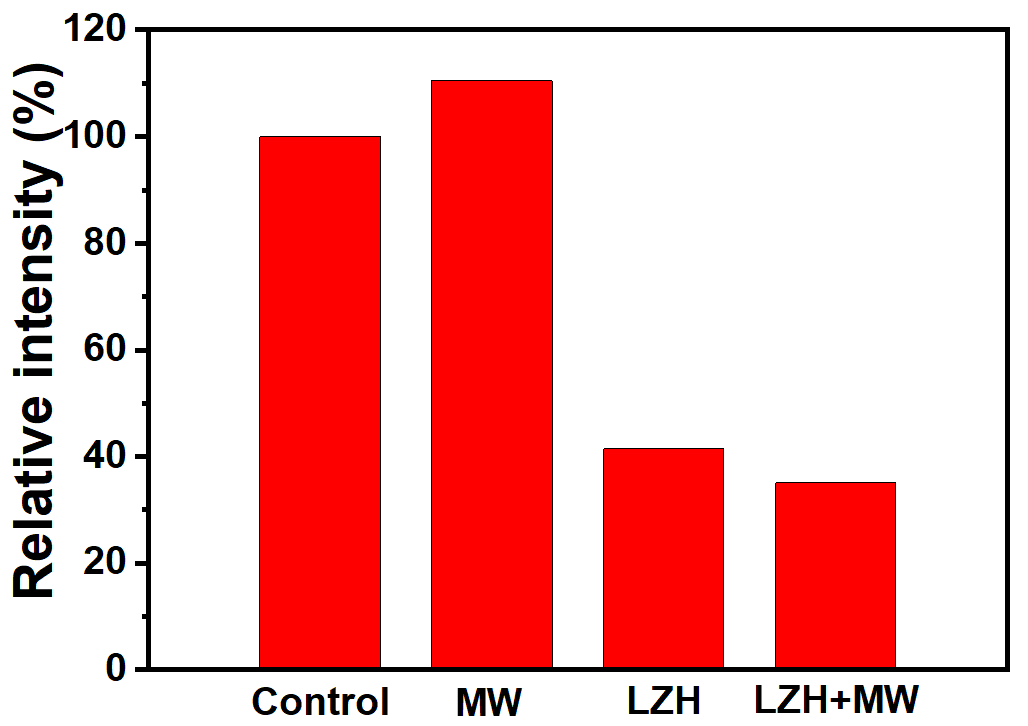


**Fig. S20** Relative intensity of immunohistochemistry.
